# Cultivable microbial diversity, peptide profiles, and bio-functional properties in Parmigiano Reggiano cheese

**DOI:** 10.3389/fmicb.2024.1342180

**Published:** 2024-03-19

**Authors:** Serena Martini, Laura Sola, Alice Cattivelli, Marianna Cristofolini, Valentina Pizzamiglio, Davide Tagliazucchi, Lisa Solieri

**Affiliations:** ^1^Nutritional Biochemistry, Department of Life Sciences, University of Modena and Reggio Emilia, Reggio Emilia, Italy; ^2^Microbial Biotechnologies and Fermentation Technologies, Department of Life Sciences, University of Modena and Reggio Emilia, Modena, Italy; ^3^Lactic Acid Bacteria and Yeast Biotechnology, Department of Life Sciences, University of Modena and Reggio Emilia, Reggio Emilia, Italy; ^4^Consorzio del Formaggio Parmigiano Reggiano, Reggio Emilia, Italy

**Keywords:** Parmigiano Reggiano cheese, *Lacticaseibacillus*, bioactive peptides, natural whey starter, peptidomics, starter lactic acid bacteria, non-starter lactic acid bacteria

## Abstract

**Introduction:**

Lactic acid bacteria (LAB) communities shape the sensorial and functional properties of artisanal hard-cooked and long-ripened cheeses made with raw bovine milk like Parmigiano Reggiano (PR) cheese. While patterns of microbial evolution have been well studied in PR cheese, there is a lack of information about how this microbial diversity affects the metabolic and functional properties of PR cheese.

**Methods:**

To fill this information gap, we characterized the cultivable fraction of natural whey starter (NWS) and PR cheeses at different ripening times, both at the species and strain level, and investigated the possible correlation between microbial composition and the evolution of peptide profiles over cheese ripening.

**Results and discussion:**

The results showed that NWS was a complex community of several biotypes belonging to a few species, namely, *Streptococcus thermophilus*, *Lactobacillus helveticus*, and *Lactobacillus delbrueckii* subsp. *lactis*. A new species-specific PCR assay was successful in discriminating the cheese-associated species *Lacticaseibacillus casei*, *Lacticaseibacillus paracasei*, *Lacticaseibacillus rhamnosus*, and *Lacticaseibacillus zeae*. Based on the resolved patterns of species and biotype distribution, *Lcb. paracasei* and *Lcb. zeae* were most frequently isolated after 24 and 30 months of ripening, while the number of biotypes was inversely related to the ripening time. Peptidomics analysis revealed more than 520 peptides in cheese samples. To the best of our knowledge, this is the most comprehensive survey of peptides in PR cheese. Most of them were from β-caseins, which represent the best substrate for LAB cell-envelope proteases. The abundance of peptides from β-casein 38–88 region continuously increased during ripening. Remarkably, this region contains precursors for the anti-hypertensive lactotripeptides VPP and IPP, as well as for β-casomorphins. We found that the ripening time strongly affects bioactive peptide profiles and that the occurrence of *Lcb. zeae* species is positively linked to the incidence of eight anti-hypertensive peptides. This result highlighted how the presence of specific LAB species is likely a pivotal factor in determining PR functional properties.

## 1 Introduction

**Figure 1:**
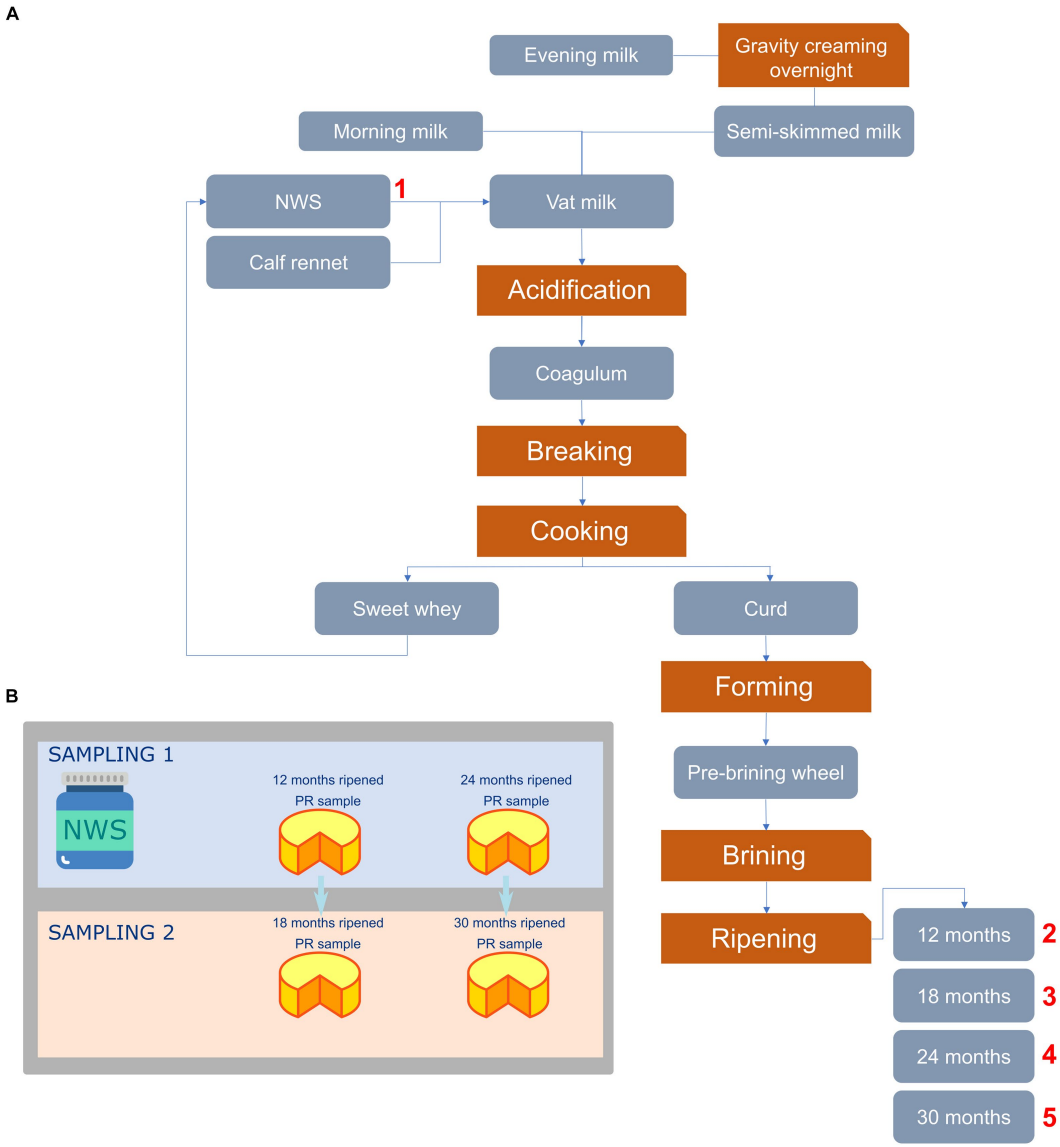
Flowchart depicting the PR cheese manufacturing process **(A)** and sampling strategy used in this study **(B)**. In panel **(A)**, numbers indicate sampling points. NWS, Natural whey starter.

Artisanal cheeses are man-driven ecosystems inhabited by composite microbial communities that originate from various sources (Bokulich and Mills, 2013; Wolfe and Dutton, 2015; Ercolini, 2020). During the process of cheese-making and ripening, biotic, and abiotic factors affect the course of microbial community evolution (Mayo et al., 2021). As a result, differences in microbial species composition affect the organoleptic and rheological attributes of the final products. This is especially true for the Parmigiano Reggiano (PR) cheese, the most famous Italian long-ripened (at least 12 months) hard-cooked cheese produced according to the specifications of the Protected Designation of Origin (PDO).^1^ PR cheese manufacturing entails the usage of raw cow milk (2.2–2.5% fat), which is a mixture of evening milk (partially skimmed by natural creaming) and morning whole milk, without adding any industrial starters or adjunct cultures (Figure 1A). The only admitted starter is the natural whey starter (NWS), produced in every dairy by incubating the whey of the previous cheese-making round at a decreasing temperature after curd cooking. The microbiota inhabiting raw milk is rapidly replaced by NWS homofermentative and thermophilic starter lactic acid bacteria (SLAB), such as *Lactobacillus helveticus*, *Lactobacillus delbrueckii* subsp. *lactis*, and *Streptococcus thermophilus* (Rossetti et al., 2008; Bottari et al., 2010; Bertani et al., 2020; Sola et al., 2022). After curd brining, SLAB rapidly depleted in favor of non-starter lactic acid bacteria (NSLAB), mainly *Lacticaseibacillus rhamnosus* (formerly *Lactobacillus rhamnosus*), *Lacticaseibacillus paracasei* (formerly *Lactobacillus paracasei*), and *Lacticaseibacillus casei* (formerly *Lactobacillus casei*) (Coppola et al., 2000; Neviani et al., 2009; Solieri et al., 2012; Gatti et al., 2014). These mesophilic and facultatively heterofermentative species belong to the so-called *Lcb. casei* group (LCG) and are supposed to arise from raw cow milk and dairy environment (Bottari et al., 2018). LCG becomes dominant starting from 2 months of ripening (Bottari et al., 2018; Bettera et al., 2023), due to their tolerance toward pH values as low as 4.9, salt concentrations up to 6%, and a wide range of temperatures (2–53°C) (Settanni and Moschetti, 2010; Gobbetti et al., 2015).

The succession of SLAB and NSLAB during PR cheese-making and ripening assures biochemical reactions that successfully transform milk/curd into ripened cheese. SLAB present in NWS rapidly turn lactose into lactic acid, enabling gel syneresis, whey expulsion, and curd formation (Wilkinson and LaPointe, 2020). Together with rennet and milk proteases, SLAB contribute to casein hydrolysis. Accordingly, several SLAB (e.g., *L. helveticus*) are highly proteolytic species (Griffiths and Tellez, 2013). During cheese manufacturing, both abiotic (e.g., carbon starvation, curd cooking, and salt levels) and biotic conditions (e.g., prophage induction) trigger SLAB autolysis, which releases intracellular peptidases and cell-envelope proteases (CEP) into cheese matrices, further contributing to proteolysis (Gatti et al., 2008). SLAB autolysis also provides carbon skeletons and nitrogen sources to support NSLAB growth (Adamberg et al., 2005; Dea Lindner et al., 2008; Lazzi et al., 2014; Sgarbi et al., 2014; Czárán et al., 2018; Møller et al., 2020). NSLAB affect cheese flavoring compounds through three metabolic pathways: (i) lactate and citrate metabolism, (ii) free fatty acid release and their subsequent metabolism, and (iii) protein breakdown and amino acid catabolism. It is well known that several NSLAB strains possess a comprehensive proteolytic system (as reviewed by Tagliazucchi et al., 2019), which is functionally active in cheese (Bove et al., 2012; Randazzo et al., 2021; Solieri et al., 2022). NSLAB isolated from PR cheese release casein-derived bioactive peptides and oligopeptides shorter than 20 aa, which exert anti-hypertensive, antioxidant, and antidiabetic activities (Tagliazucchi et al., 2020). Therefore, the extent of proteolysis carried out by intact NSLAB cells and their released enzymes strongly contribute to the rheological, organoleptic, and functional attributes of PR cheese (Martini et al., 2020, 2021; Solieri et al., 2020; Tagliazucchi et al., 2020).

Based on the importance of SLAB and NSLAB in PR cheese-making, this study aimed to explore the cultivable microbial fraction in NWS and PR wheels at different ripening times (namely 12, 18, 24, and 30 months) and to determine the evolution of peptidomic profiles over time. Possible correlations between the observed microbial diversity, the peptidomic profiles, and the related bio-functional properties were unraveled.

## 2 Materials and methods

### 2.1 Sampling

In the present study, a total of three NWS and 12 cheese samples were collected from February 2020 to January 2021 from three different dairies located in the province of Reggio Emilia (Italy) and belonging to the Parmigiano Reggiano Cheese Consortium. For each dairy, the sampling flowchart is shown in Figure 1. Specifically, we collected three NWS as well as 12 and 24 months-ripened PR cheese samples in the first sampling. The second sampling was carried out after 6 months, collecting PR cheese samples from the same wheels after 18 and 30 months of ripening. Samples were aseptically handled and immediately brought into the laboratory under refrigerated conditions for the subsequent analysis. Each sample name consisted of a letter (corresponding to the respective dairy farm) and a number (corresponding to the ripening time).

### 2.2 Materials and type strains

All media and chemicals were purchased from Oxoid (Basingstoke, Hampshire, United Kingdom) and Sigma-Aldrich (St. Louis, MO, United States), respectively, except where differently indicated. Anaerobic systems and molecular biology reagents were purchased from Thermo Fisher Scientific (Waltham, MA, United States). Oligonucleotide and Sanger sequencing services were provided by Bio-Fab Research (Rome, Italy).

The type strains used in this study are listed in Table 1. All the strains were purchased from DSMZ (Deutsche Sammlung von Mikroorganismen und Zellkulturen GmbH, Deutschland) and cultivated according to DSMZ culture condition specifications.

**Table 1:** Reference strains used in this study.

Strains	Species	Culture conditions
DSM20011^T^	*Lacticaseibacillus casei*	MRS pH 6.2–6.5; 48 h; 37°C; anaerobiosis
DSM 5622^T^	*Lacticaseibacillus paracasei* subsp. *paracasei*	MRS pH 6.2–6.5; 48 h; 37°C; anaerobiosis
DSM20021^T^	*Lacticaseibacillus rhamnosus*	MRS pH 6.2–6.5; 48 h; 37°C; anaerobiosis
DSM20178^T^	*Lacticaseibacillus zeae*	MRS pH 6.2–6.5; 48 h; 37°C; anaerobiosis
DSM20617^T^	*Streptococcus salivarius* subsp*. thermophilus*	MRS pH 6.2–6.5; 48 h; 37°C; anaerobiosis
DSM20075^T^	*Lactobacillus helveticus*	MRS pH 6.2–6.5; 48 h; 37°C; anaerobiosis
DSM 20052^T^	*Limosilactobacillus fermentum*	MRS pH 6.2–6.5; 48 h; 37°C; anaerobiosis
DSM20074^T^	*Lactobacillus delbrueckii* subsp. *delbrueckii*	MRS pH 6.2–6.5; 48 h; 37°C; anaerobiosis
DSM20081^T^	*Lactobacillus delbrueckii* subsp. *bulgaricus*	MRS pH 6.2–6.5; 48 h; 37°C; anaerobiosis

### 2.3 Physicochemical characterization of NWS and compositional analysis of cheeses

Natural whey starter samples were analyzed for pH and titratable acidity as previously reported (Sola et al., 2022). Concerning cheese samples, after removing 15 mm of the rind, cheese cores were finely shredded and immediately analyzed for moisture, NaCl, protein, and fat contents, according to Lolli et al. (2021). NaCl/DM (dry matter), protein/DM, and fat/DM were also calculated. All analyses were performed using the NIRFlex N-500 (Büchi Labortechnik AG, Flawil, Switzerland) working with near infrared reflection (NIR) in the region 800–2,500 nm. The analyses were carried out in triplicate.

### 2.4 Microbiological counts and LAB isolation

To prepare the cheese samples, 5 g of each cheese sample from the medial section of the core (the length varied between 18 and 22 cm) were aseptically transferred into a sterile stomacher bag, supplemented with 45 mL of sterile saline solution (9 g/L NaCl), and homogenized for 4 min at 220 rpm in a Stomacher LAB Blender 400 (PBI International, Milan, Italy). NWS samples were 10-fold diluted with sterile saline solution (9 g/L NaCl) before plating. Enumerations of LAB populations were carried out for NWS and cheese samples using de Man, Rogosa, and Sharpe (MRS) agar medium at a pH of 5.4, incubated at 37°C for 72 h under anaerobic conditions, and M17 medium, incubated at 42°C for 72 h under anaerobiosis. The NWS M17 medium supplemented with 7% (v/v) of sterile skimmed milk (SSW) (Morga AG, Ebnat-Kappel, Switzerland) was prepared and incubated at 42°C for 72 h under aerobiosis, as described by Fornasari et al. (2006). All media were added with the antibiotic cycloheximide (100 mg/L) to inhibit yeasts. Viable cell counts were recorded as a number of colony-forming units (CFU)/mL recovered from plates with CFU/mL ranging from 20 to 200 and expressed as Log_10_ CFU/mL means of at least three replicates.

Individual bacterial colonies were randomly selected, sub-cultured on the same isolation medium, screened for catalase reaction and Gram staining, and microscopically checked before storing at −80°C in liquid culture using 25% (v/v) of glycerol solution.

### 2.5 LAB identification

Genomic DNA was obtained through mechanical lysis of bacterial cells in the late exponential phase and organic solvent extraction as previously described (Tagliazucchi et al., 2020). The purity and quantity of DNA were estimated using a NanoDrop ND-1000 spectrophotometer (NanoDrop Technologies, Wilmington, DE, United States). The DNA quality was also measured by electrophoresis on 0.8% (w/v) agarose gel containing ethidium bromide (0.5 mg/mL) in 0.5× TBE buffer (45 mmol/L Tris–HCl, 45 mmol/L boric acid, and 1 mmol/L EDTA, with a pH of 8.0) at a constant voltage of 90 V for 1 h at room temperature. Gel pictures were taken under UV light using a BioDoc analysis system (Biometra, Göttingen, Germany). Finally, the DNA samples were diluted to 50 ng/μL in ddH_2_O and were stored at −20°C for subsequent analyses.

Natural whey starter isolates were identified by pentaplex PCR as previously reported (Cremonesi et al., 2011), except for primer concentrations reduced to 1 μmol/L for each primer. Cheese isolates were identified by *mutL* gene multiplex PCR targeting the species *Lacticaseibacillus casei*, *Lacticaseibacillus paracasei*, and *Lacticaseibacillus rhamnosus* (Bottari et al., 2017), with the following improvement. A fourth target species, *Lacticaseibacillus zeae*, was implemented in the multiplex PCR assay using a primer pair designed on gene KRK10099.1 [locus_tag = “FD51_GL001918” on *Lcb. zeae* DSM20168^T^ genome (AZCT01000025.1:29282.30181), namely, Zeae_F1 (5′-TTTGACCGGTTAGATGACCAGCAT-3′) and Zeae_R1 (5′-CGCGACATGTTGGTAAGGTGCG-3′)]. A comprehensive list of primers and PCR cycling conditions used in this study is reported in Supplementary Table S1. All PCRs were carried out using a thermal cycler T100 (Bio-Rad, Hercules, CA, United States) in a final volume of 20 μL containing DreamTaq Green Buffer 1 × 2 mmol [L of MgCl_2_, 200 μmol/L of each dNTP, 50 ng of DNA template, 0.5 U DreamTaq Green DNA polymerase (5 U/μL), and 0.5 μmol/L of each primer in multiplex PCR and 1 μmol/L of each primer in pentaplex PCR, respectively]. When required, 16S rRNA gene PCR amplification and ARDRA analysis with diagnostic endonucleases, such as *Mse*I, *EcoR*I, and *Hha*I, were carried out for NWS and cheese isolates, respectively (Sola et al., 2022).

### 2.6 Phylogenetic analysis

PCR amplicons of the 16S rRNA gene were purified using the DNA Clean & Concentrator™-5 Kit (Zymo Research, Orange, CA, United States) and were sequenced on both strands using 27f and 1,490 primers through a DNA Sanger dideoxy sequencing process performed by Bio-Fab Research (Rome, Italy). When required, internal primer WLAB2 (5′-TCGAATTAAACCACATGCTCCA-3′) (Lopez et al., 2003) was also used for sequencing. Contig sequences were merged using the program SeqMan (DNASTAR, Madison, WI, United States), and the poor-quality ends were edited manually to remove primers. The resulting contig sequences were used as queries in a Blastn search against the NCBI RefSeq database (O'Leary et al., 2016) (14 September 2023). A cutoff of 98.7% 16S rRNA gene similarity was used for species attribution (Stackebrandt, 2006). The related sequences, i.e., four outgroup species (*Weizmannia coagulans*, *Bacillus subtilis*, *Bacillus vallismortis*, and *Enterococcus faecalis*) (Ventura et al., 2009), were aligned with the Muscle program (Edgar, 2004) in Mega X software (Kumar et al., 2018), and the resulting alignment was subjected to a DNA substitution model analysis to select the best-fitting model. Phylogenetic relationships were inferred using the maximum likelihood method. Among sites, rate variation was modeled by a gamma distribution (+G). Bootstrap support values were obtained from 1,000 random resamplings. Trees were visualized using the Interactive Tree of Life (ITOL) (Letunic and Bork, 2019) and were rooted at outgroup reference species. The sequences obtained in this study were deposited in the GenBank NCBI database with the accession numbers ON936796–ON936814 and OM091849–OM091851.

### 2.7 rep-PCR and fingerprinting analysis

Genotyping of LAB isolates was performed by the repetitive sequence-based polymerase chain reaction (rep-PCR) using marker (GTG)_5_ (5′-GTGGTGGTGGTGGTG-3′), as previously described (Tagliazucchi et al., 2020). Fingerprinting patterns were analyzed using BioNumerics software v8.10 (Applied Maths, Sint-Martens-Latem, Belgium). A unique dataset was used to analyze 65 SLAB. For 189 NSLAB submitted to UGMA analysis, we created four datasets based on the ripening time, namely, 12_m (12 months of ripening), 18_m (18 months of ripening), 24_m (24 months of ripening), and 30_m (30 months of ripening). Particularly, the Pearson correlation coefficient was used to calculate similarity matrices from densitometric curves. To define appropriate parameters (optimization and curve smoothing), we used the software’s optimization tools, which build dendrograms to identify distinct isolates and conduct bootstrap replications to test a range of possible values and find the most parsimonious branching solution. Clustering analysis of similarity matrices was performed using the unweighted pair-group method with arithmetic mean (UPGMA) algorithm with 1,000 bootstrapping replicates to evaluate the consistency of the group. The resulting trees were visualized using ITOL, as reported above. LAB isolates displaying a similarity greater than 91% were considered to have the same biotype.

### 2.8 Extraction of low-molecular-weight water-soluble peptides from PR cheese samples

Water-soluble peptides were first extracted from PR cheeses by following the protocol reported in Martini et al. (2021). Briefly, 5 g of cheese were mixed with 45 mL of 0.1 mol/L HCl, and the mixture was homogenized using an Ultra-Turrax homogenizer. Three cycles of homogenization lasting 1 min were carried out, alternated with 1 min in an ice bath. Subsequently, the homogenates were centrifuged (40 min; 4°C; 4,000 *g*) and then filtered using Whatman filter paper 4. The clear water-soluble peptide extracts were then subjected to ultrafiltration to get the low-molecular-weight peptide fractions for peptidomics analysis. Ultrafiltration was carried out with a membrane of 3 kDa cutoff, as previously described (Tagliazucchi et al., 2017).

### 2.9 Peptides identification and semi-quantitative analysis

Low-molecular-weight peptide fractions were analyzed via the peptidomics technique for outlining PR cheese peptide profiles from a qualitative and semi-quantitative point of view. The samples were injected into a UHPLC system (UHPLC UltiMate 3000 separation module, Thermo Scientific, San Jose, CA, United States) coupled with a high-resolution mass spectrometry (Q Exactive^™^ Plus Hybrid Quadrupole-Orbitrap^™^ Mass Spectrometer, Thermo Scientific, San Jose, CA, United States). The complete details about the chromatographic conditions, mass spectrometry, and tandem mass spectrometry parameters were reported in Martini et al. (2021). Qualitative and semi-quantitative analyses were carried out using Mascot and Skyline software, respectively, as previously described (MacLean et al., 2010; Dallas and Nielsen, 2018; Martini et al., 2021; Helal et al., 2023).

Bioactive peptide identification was performed using the Milk Bioactive Peptide Database, considering only the peptides with 100% sequence homology with previously characterized bioactive peptides (Nielsen et al., 2017).

### 2.10 Statistical analysis

Significant differences among samples were evaluated by one-way ANOVA with Tukey post-test. Data were considered significantly different when *p* < 0.05. Three analytical replicates for each sample were used for all analyses.

The semi-quantitative bioactive peptide data of the cheese samples were utilized for chemometric analysis by using online software MetaboAnalyst 5.0 (Xia et al., 2015) (REF). Data were normalized by median and Pareto scaling before principal component analysis (PCA) and partial least squares discriminant analysis (PLS-DA). The analysis was validated by multiple correlation coefficients (R2) and cross-validation (Q2). The significance of the biomarkers was ranked using the projection variable importance score (VIP score > 1) of the PLS-DA.

The correlation among the variables (semi-quantitative bioactive peptide data, microbiological data, and compositional data) was assessed by Spearman rank analysis (*p* < 0.05) with MetaboAnalyst 5.0.

## 3 Results

### 3.1 NWS physicochemical characterization and microbial counts

In this study, three NWS samples from three manufacturers in the Italian province of Reggio Emilia, referred to as R, C, and L, were considered. The samples NWS_R, NWS_C, and NWS_L showed pH values of 3.46 ± 0.01, 3.46 ± 0.05, and 3.49 ± 0.04, respectively. Titratable acidity ranged from 27.29 ± 0.05 to 29.90 ± 0.04, while lactic acid concentrations were from 12.29 ± 0.21 to 13.48 ± 0.28 (Supplementary Table S2).

**Figure 2:**
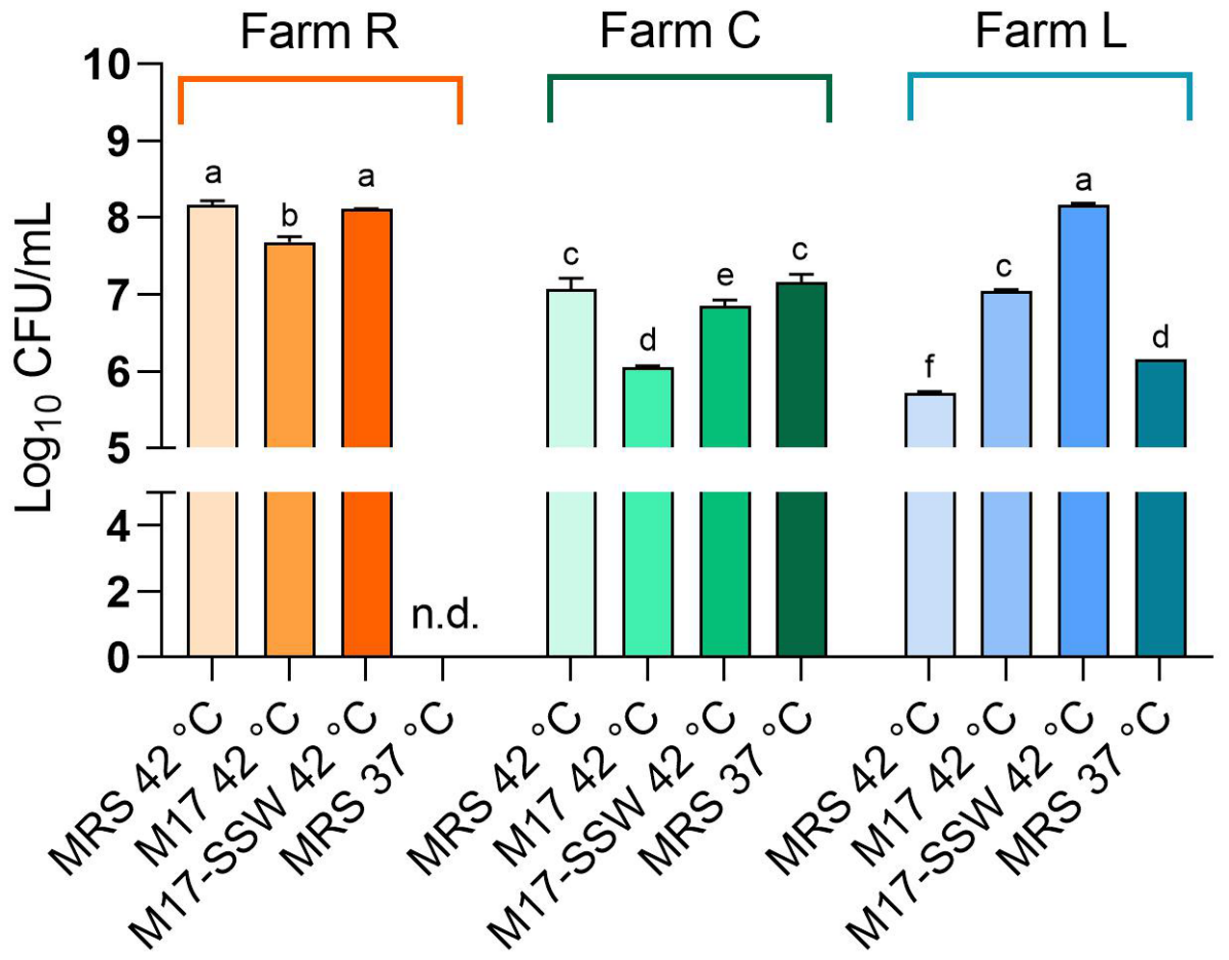
Starter lactic acid bacteria (SLAB) plate counts from NWS samples. Data from dairies R (orange), C (green), and L (blue) are mean (*n* = 3) of Log_10_ CFU/mL values, while error bars represent standard deviations. Different letters indicate significant differences among samples and conditions (*p* < 0.05). nd—, Not determined.

To detect the broadest spectrum of NWS cultivable fraction as possible, we used four different growth conditions. Microbial counts are reported in Figure 2. The SLAB counts ranged from 8.17 ± 0.05 (NWS_R, MRS at 42°C) to 5.72 ± 0.02 (NWS_L, MRS at 42°C) Log_10_ CFU/mL. NWS_R generally had higher counts of SLAB than NWS_C and NWS_L in all growth conditions tested (*p* < 0.05). The only exception was SLAB counts in M17-SSW medium from dairy L, which were similar to those scored in R (MRS 42°C; M17-SSW 42°C).

### 3.2 SLAB diversity and species distribution

**Figure 3:**
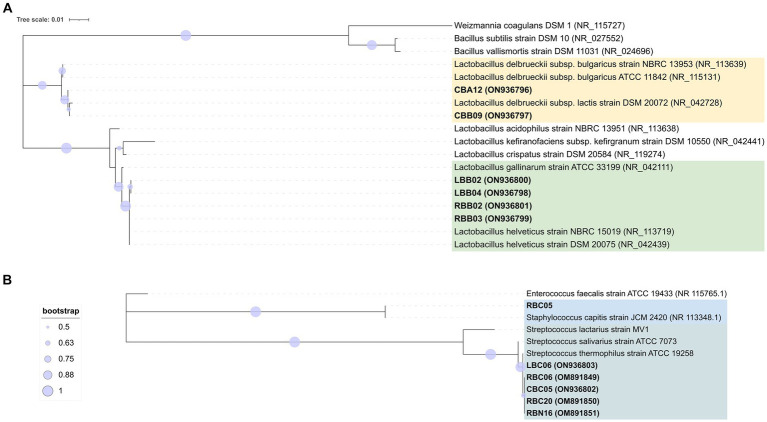
Phylogenetic trees based on 16S rRNA gene sequences showing the relationship among rod-shape **(A)** and cocci **(B)** SLAB strains isolated from three NWS samples and the related neighbor’s species. The trees were inferred using the maximum likelihood method and the Kimura’s two-parameter model (Kimura, 1980) with Mega X software (Kumar et al., 2018). Representative isolates for each sample are shown with the sequence accession numbers indicated in brackets, while the sequence data of reference strains were from the NCBI RefSeq database. A discrete Gamma distribution was used to model evolutionary rate differences among sites. Bootstrap values are indicated at branch points based on 1,000 replications. Bootstrap values below 50% are not shown. Bar: 0.01 substitutions per nucleotide position. The trees are drawn in scale, with branch length measured in the number of substitutions per site. The trees were rooted using the branch leading to three outgroup species: *W. coagulans*, *B. subtilis*, and *B. vallismortis* for the panel **(A)** and *E. faecalis* for the panel **(B)**.

A total of 65 SLAB isolates were submitted to species attribution, which entailed pentaplex PCR and 16S ARDRA analysis with endonucleases *Mse*I and, in case of *L. delbrueckii*, 16S ARDRA with *EcoR*I. Details on molecular identification results are reported in Supplementary Table S3. Both approaches were consistent in attributing 53.85% of isolates to *S. thermophilus* species, followed by 27.69% to *L. helveticus* and 16.92% to *L. delbrueckii* subsp. *lactis* (16.92%). The remaining 1.54% of isolates did not give any results with pentaplex PCR, while 16S ARDRA analysis resulted in a restriction pattern A that did not match those exhibited by the type strains considered in this study. At least one strain for each sample and for each 16S ARDRA profile was submitted to 16S rRNA gene partial sequencing. Two phylogenetic trees were constructed based on 16S rRNA gene sequences with their closest phylogenetic neighbors for rod-shaped and cocci isolates, respectively (Figures 3A,B, respectively). Strains CBB09 and CBA12 formed a monophyletic group with *L. delbrueckii* subsp. *lactis*, while strains RBB02, RBB03, LBB02, and LBB04 shared higher than 99% homology with *L. helveticus* NBRC1519 (Figure 3A). The analysis grouped LBC06, CBC05, RBC06, RBC20, and RBN16 with *S. thermophilus* ATCC19258^T^, while strain RBC05 was grouped separately with *Staphylococcus capitis* JCM2420 (Figure 3B).

**Figure 4:**
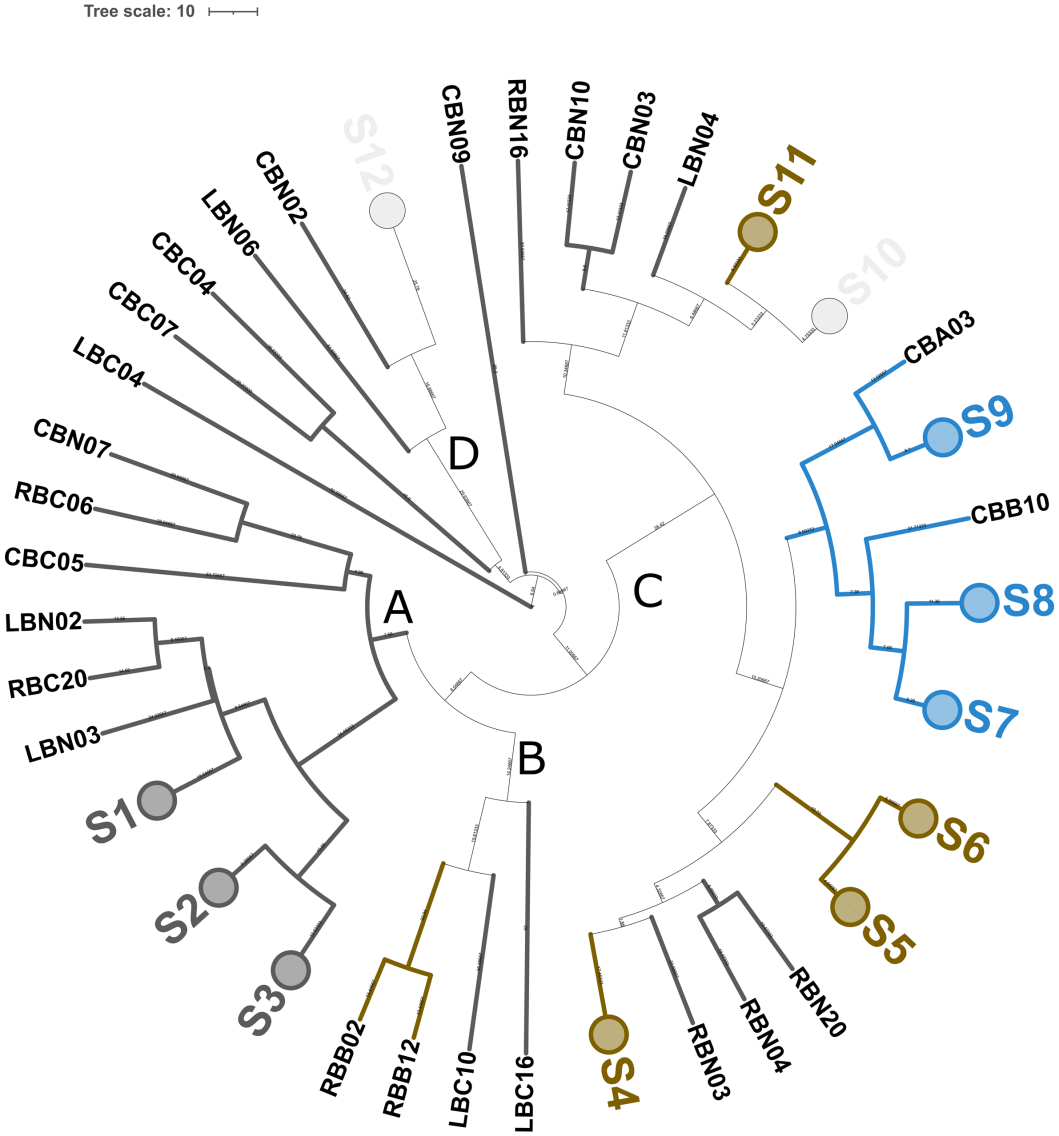
Genotyping of 65 SLAB isolates from NWS samples. Dendrogram of (GTG)_5_-PCR fingerprints was built according to the Pearson correlation coefficient and UPGMA. Collapsed clades (S1–S12) represent 12 clusters with more than 90% similarity coefficient. Upper case letters from A to D indicate major clusters. Each knot shows the length of the arms. Colors were according to the species, as follows: blue, *L. delbrueckii* subsp. *lactis*; orange, *L. helveticus*; dark gray, *St. thermophilus*; and light gray, mixed species. The tree was visualized with ITOL (Letunic and Bork, 2019).

Genotyping with microsatellite primer (GTG)_5_ resulted in 65 reliable banding patterns with band size ranging from 580 to 4420 bp. The number of amplicons ranged from 7 to 18 for each isolate. UPGMA analysis of fingerprinting data using 91% as a cutoff of reproducibility discriminated 37 biotypes, namely, 12 subclusters (S) and 25 singletons, with a discriminatory power of 0.970, as calculated by Simpson’s index of diversity (Figure 4). These 37 biotypes were grouped into four major clusters (named from A to D). Major clusters A and D grouped *S. thermophilus* strains, while B and C were promiscuous and grouped isolates belonging to at least two different species. Within the largest major cluster B, 11 *L. delbrueckii* strains were grouped congruently with their taxonomic positions and were divided into five biotypes. Additionally, 21 and 8 subclusters were found to group 35 *S. thermophilus* and 18 *L. helveticus* isolates, respectively. In a few cases (subclusters S10 and S12), *L. helveticus* and *S. thermophilus* isolates did not cluster according to their taxonomic attribution. Generally, the genetic relatedness was congruent with the sampling site, with a few exceptions (subclusters S10 and S12). According to Figure 4, dairies R, C, and L had 13, 14, and 14 biotypes, respectively.

**Figure 5:**
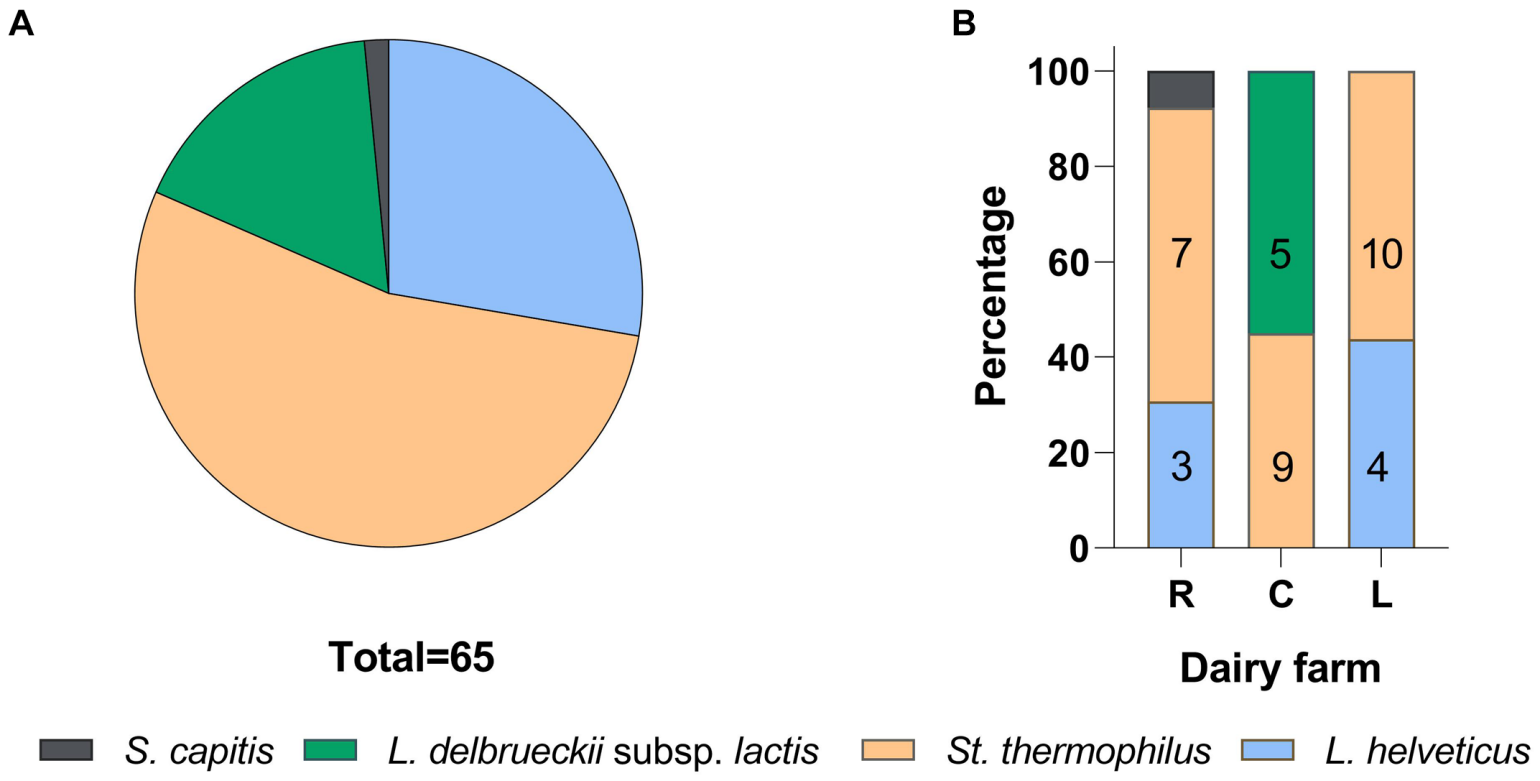
SLAB species frequencies **(A)** and distribution **(B)** in PR NSW samples. The numbers in the columns represent biotypes scored by the UPGMA analysis of (GTG)_5_ rep-PCR fingerprinting data.

Species distribution per sampling site is shown in Figure 5. *Streptococcus thermophilus* was the dominant species in dairies R and L, followed by *L. heleveticus*. In sampling site C, only *L. delbruecki* and *S. thermophilus* were detected.

### 3.3 Chemical composition and microbial counts in PR cheese

In the present study, a total of 12 PR samples were collected from the same dairy, which provided NWS samples. The ripening periods varied from 12 to 30 months. Salt, fat, protein, and moisture contents along with other parameters are reported in Supplementary Table S4. Generally, the moisture content exhibited an inverse trend compared with the ripening time.

**Figure 6:**
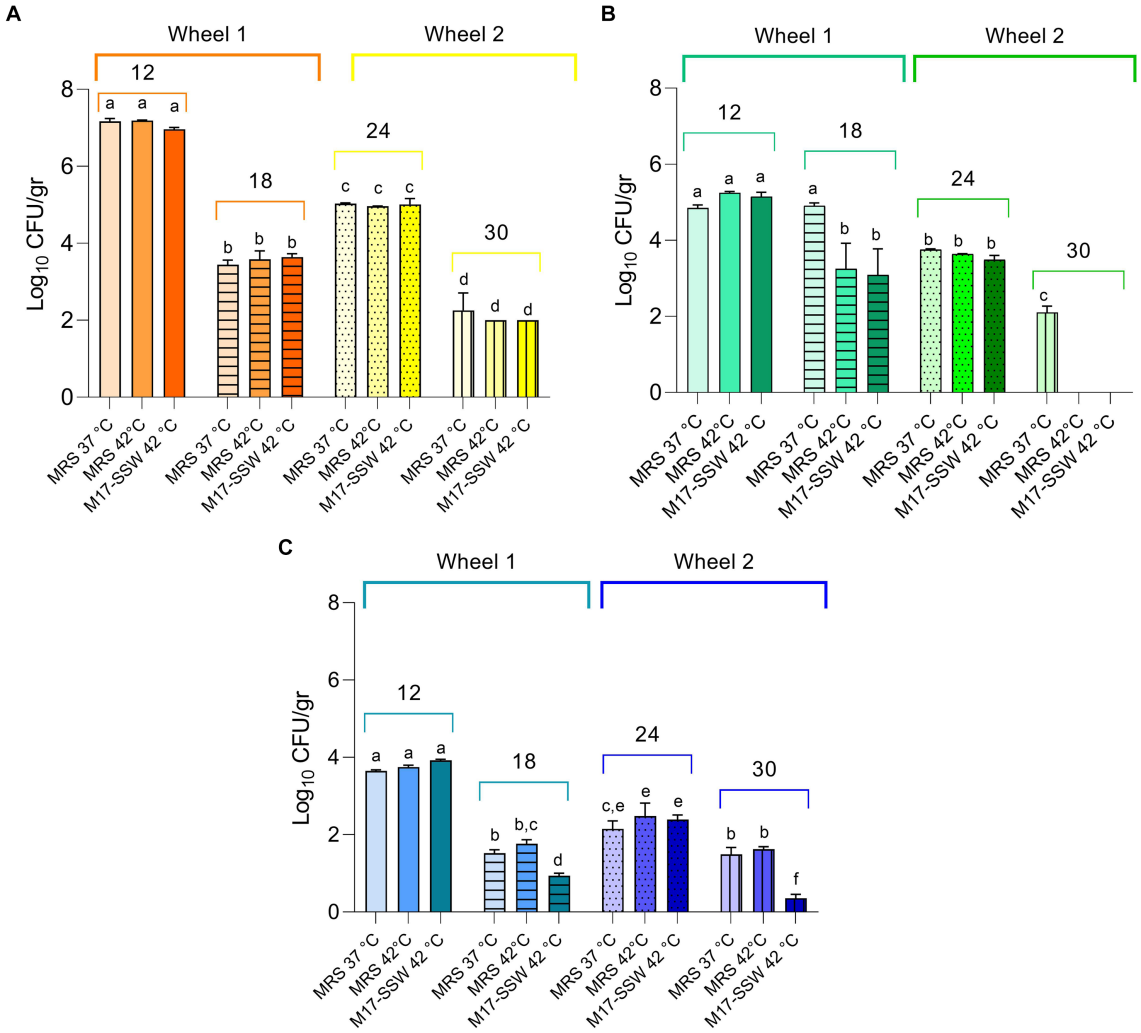
Non-starter lactic acid bacteria (NSLAB) plate counts from cheese samples. Data from dairies R (orange) **(A)**, C (green) **(B)**, and L (blue) **(C)** are mean (*n* = 3) of Log_10_ CFU/mL values, while error bars represent standard deviations. Different letters indicate significant differences among samples and conditions (*p* < 0.05).

Three different cultivation conditions were considered to recover NSLAB cultivable fractions from PR cheese samples. As shown in Figure 6, we did not find any significant differences in Log_10_ CFU/gr values among growth conditions within the same samples (*p* > 0.05), with a few exceptions. In sample C30, MRS medium at 37°C was the only condition suitable to sustain bacterial growth, while in L18 and L30, M17-SSW medium at 42°C resulted in lower Log_10_ CFU/gr compared to the other conditions. Generally, NSLAB counts ranged from 7.19 ± 0.01 to 0.36 ± 0.10 Log_10_ CFU/gr in R12 incubated in MRS at 42°C and L30 incubated in M17-SSW at 42°C, respectively (Figure 6). A general decrease in the NSLAB cultivable fraction was scored over time in all the samples (*p* < 0.05). Variations in microbial loads among wheels of the same dairy were observed (Figure 6). For instance, the C24 sample had a NSLAB population slightly higher than the C18 sample (*p* < 0.05). This could be due to the homemade nature of PR cheese, manufactured with raw cow milk and NWS. Comparing dairies with each other, the lowest Log_10_ CFU/gr values were recovered from the samples collected in dairy L in all tested conditions, whereas the highest ones were collected in all the samples from dairy R (*p* < 0.05), except for the 18-month ripening time (Figure 6). These results could mean that viable cells with an integrous cellular wall vary among samples, resulting in putative differences in releasing intracellular peptidases and in the extent of proteolysis.

### 3.4 Validation of improved multiplex PCR for NSLAB identification

Recently, Liu and Gu (2020) reestablished *Lcb. zeae* as a separate species within the LCG. Notable 16S ARDRA with *Hha*I was unable to discriminate *Lcb. zeae* DSM 20178^T^ from *Lcb. paracasei*, while conventional *mutL* multiplex PCR did not distinguish *Lcb. zeae* DSM 20178^T^ from *Lcb. casei* DSM 20011^T^ (Supplementary Figure S1A). On the other hand, Laref and Belkheir (2022) reported that seven endonucleases were required to separate LCG species. To overcome this caveat, we developed a fast PCR assay targeting a *Lcb. zeae* species-specific gene. The locus FD51_GL001918 encoding glycosyl transferase family 8 has been previously demonstrated to be present in the *Lcb. zeae* genome only (Kim et al., 2020). Therefore, it was selected as a target for designing species-specific primers. The *mutL* gene multiplex PCR assay was implemented with a new *Lcb. zeae* species-specific primer pair, resulting in an improved multiplex PCR assay suitable to discriminate *Lcb. zeae*, *Lcb. paracasei*, *Lcb. rhamnosus*, and *Lcb. casei* in a single round of PCR reaction (Supplementary Figures S1B,C).

### 3.5 NSLAB diversity and species distribution

**Figure 7:**
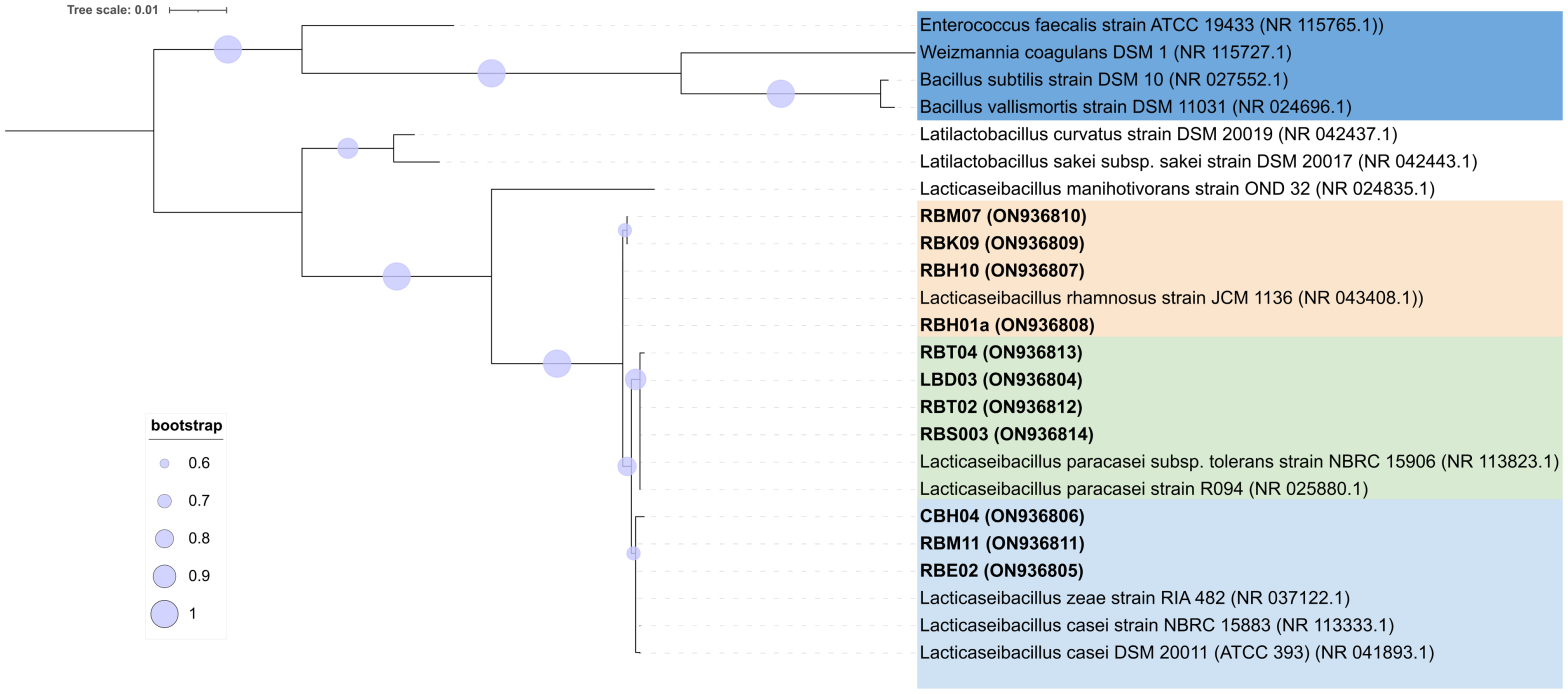
Phylogenetic tree based on 16S rRNA gene sequences showing the relationships among non-starter lactic acid bacteria (NSLAB) strains isolated from PR cheeses at 12, 18, 24, and 30 months of ripening. The tree was constructed using the maximum likelihood method and the Kimura’s two-parameter model (Kimura, 1980) in Mega X software. Representative isolates for each sample are shown in bold, with the sequence accession numbers indicated in brackets. Sequence data for reference strains were from the NCBI RefSeq database. A discrete Gamma distribution was used to model evolutionary rate differences among sites. The final dataset involved 24 nucleotide sequences for a total of 1,372 positions. Bootstrap values (>60%) are indicated at branch points based on 1,000 replications. The tree is rooted using the branch leading to four outgroup species (*W. coagulans*, *B. subtilis*, *B. vallismortis*, and *E. faecalis*). The tree is drawn in scale with branch length measured in the number of substitutions per site: bar, 0.01 substitutions per nucleotide position. The outgroup species are highlighted in the blue background, *Lcb. rhamnosus* in orange background, *Lcb. casei*/*Lcb. zeae* in green background, and *Lcb. paracasei* in light blue background, respectively. The tree was visualized with ITOL (Letunic and Bork, 2019).

Two hundred and fifteen Gram-positive and catalase-negative isolates were obtained from PR cheeses at 12, 18, 24, and 30 months, respectively. An overview of isolates and details on molecular species attribution carried out with 16S ARDRA and improved multiplex PCR assay are reported in Supplementary Table S5. Finally, sequencing of the 16S rRNA gene and phylogenetic analysis were used to confirm species attribution based on 16S ARDRA and improved multiplex PCR assay (Figure 7). Based on the adopted polyphasic approach, 105 isolates were ascribed to *Lcb. paracasei*, 67 to *Lcb. zeae*, 42 to *Lcb. rhamnosus*, and 1 isolate to *Lcb. casei*.

**Figure 8:**
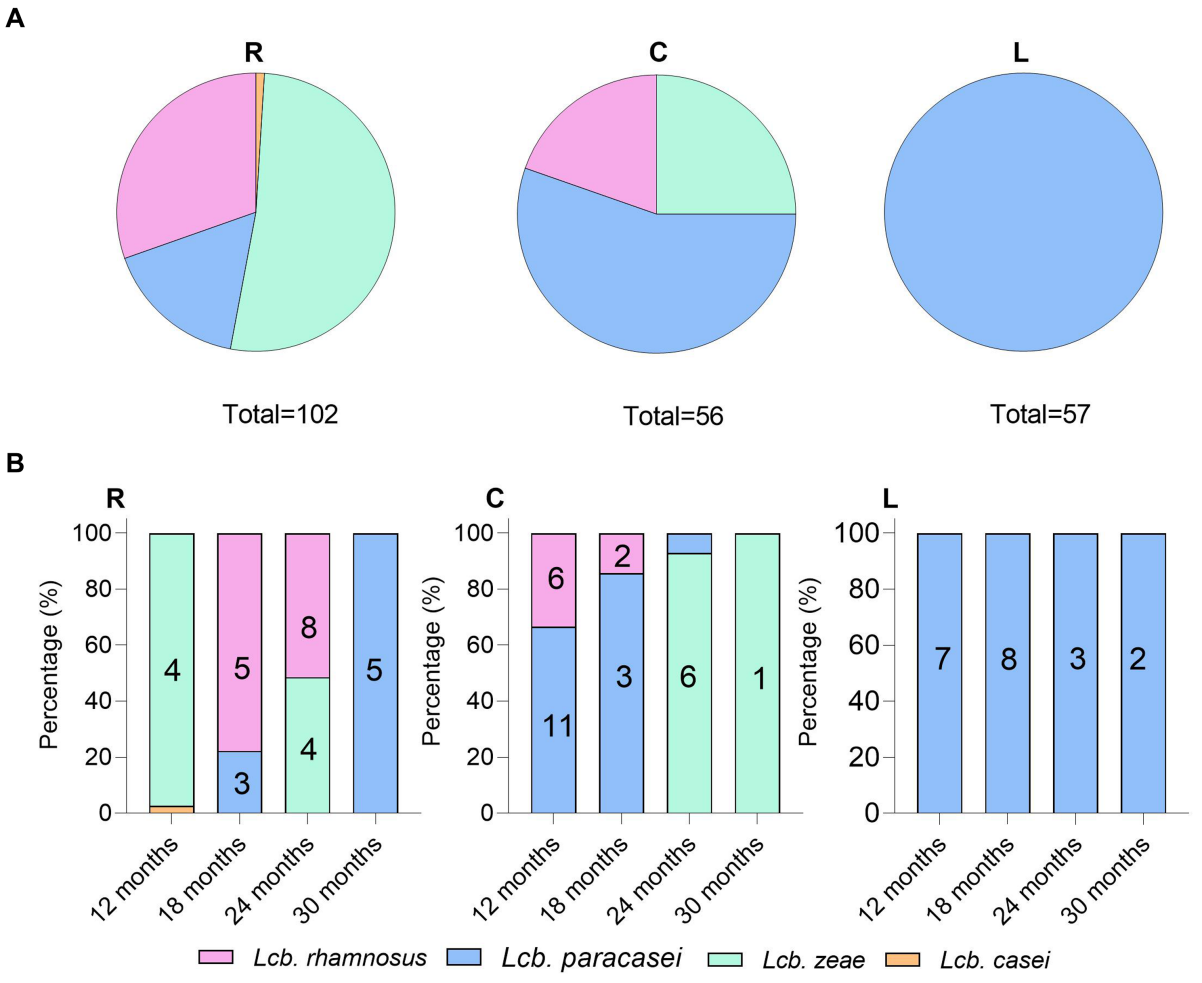
Non-starter lactic acid bacteria (NSLAB) species frequencies **(A)** and distribution **(B)** in PR cheese samples. The numbers in the columns represent biotypes scored by UPGMA analysis of (GTG)_5_ rep-PCR fingerprinting data.

Analysis of species frequencies showed that species patterns were strictly dairy-dependent (Figure 8A). NSLAB populations inhabiting ripened PR wheels reflect the quality and microbiological variability of raw cow milk, resulting in a strong dairy-to-dairy variation in species composition. While *Lcb. paracasei* was ubiquitous, *Lcb. zeae* and *Lcb. rhamnosus* were differently recovered depending on the sampling site. Dairy R was positive for all four species belonging to the LCG clade; dairy C was positive for *Lcb. zeae*, *Lcb. rhamnosus*, and *Lcb. paracasei*. Finally, *Lcb. paracasei* was the only species recovered in dairy L (Figure 8A). We observed that the recovery of *Lcb. rhamnosus* decreased with increasing ripening time. *Lcb. paracasei* dominated dairy R samples, regardless of the ripening time (Figure 8B), while in sampling site C, *Lcb. zeae* became dominant starting after 24 months of ripening (Figure 8B).

**Figure 9:**
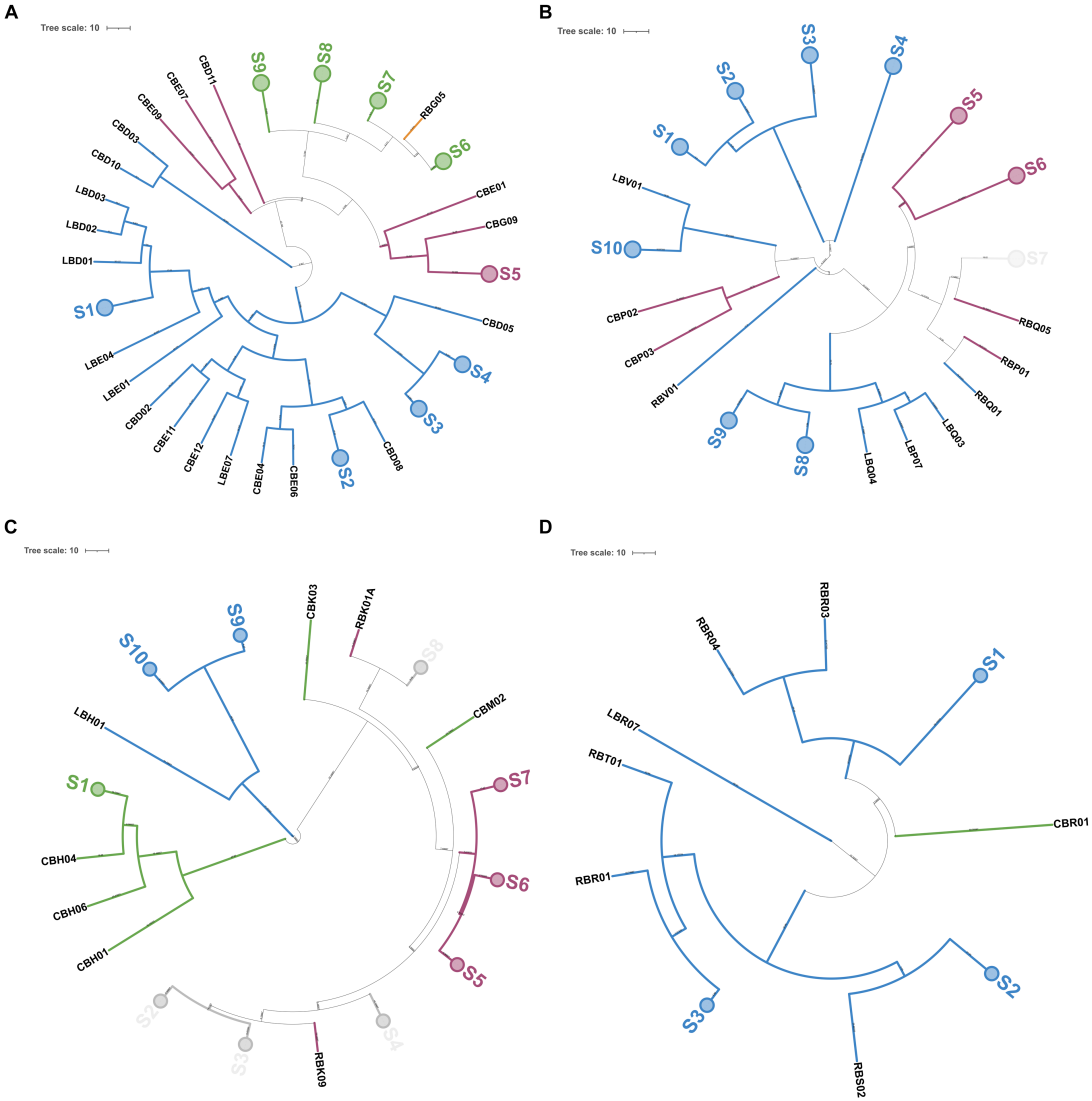
Genotyping of 189 NSLAB isolates from PR cheese samples. Trees **(A–D)** represent NSLAB isolates from 12, 18, 24, and 30 months of ripening, respectively. Dendrograms of (GTG)_5_-PCR fingerprints were built according to the Pearson correlation coefficient and UPGMA. In each tree, collapsed clades (S) represent clusters with more than 91% similarity coefficient. Each knot shows the length of the arms. Colors were according to the species, as follows: blue, *Lcb. paracasei*; pink, *Lcb. rhamnosus*; green, *Lcb. zeae*; and light gray, mixed group. The tree was visualized with ITOL (Letunic and Bork, 2019).

Out of 215 NSLAB identified at the species level, 189 resulted in reliable banding patterns by rep-PCR with microsatellite primer (GTG)_5_. The number of amplicons ranged from 7 to 18 for each pattern. The UPGMA analysis of fingerprinting data was carried out using four datasets; each of them contained isolates belonging to the same ripening time (12, 18, 24, and 30_m, respectively). Figure 9 shows the resulting UPGMA clustering trees. When 91% reproducibility cutoff was used, 68 NSLAB isolates from 12-month ripened PR cheeses were grouped into 30 biotypes (9 subclusters and 21 singletons) (Figure 9A); 48 isolates from the 18_m dataset were divided into 20 biotypes (10 subclusters and 10 singletons) (Figure 9B); 59 isolates from 24_m into 18 biotypes (10 subclusters and 8 singletons) (Figure 9C); and 18 isolates from 30_m into 10 biotypes (3 subclusters and 7 singletons) (Figure 9D). The Simpson’s indices of diversity were 0.921, 0.924, 0.915, and 0.895 for 12, 18, 24, and 30_m, respectively, suggesting that biodiversity slightly decreased with increase in the ripening time and selective pressure. In most cases, biotypes are grouped congruently with species attribution and sampling site. The only exceptions were the subclusters S2, S3, S4, and S8 in the 24_m dataset, where *Lcb. zeae* and *Lcb. rhamnosus* formed mixed subclusters (Figure 9C), as well as subcluster S7 in the 18_m dataset where *Lcb. rhamnosus* and *Lcb. paracasei* isolates were intermixed (Figure 9B). According to Figure 8, dairies R, C, and L showed a high number of biotypes.

### 3.6 Peptidomics analysis of PR cheeses and bioactive peptides identification

**Figure 10:**
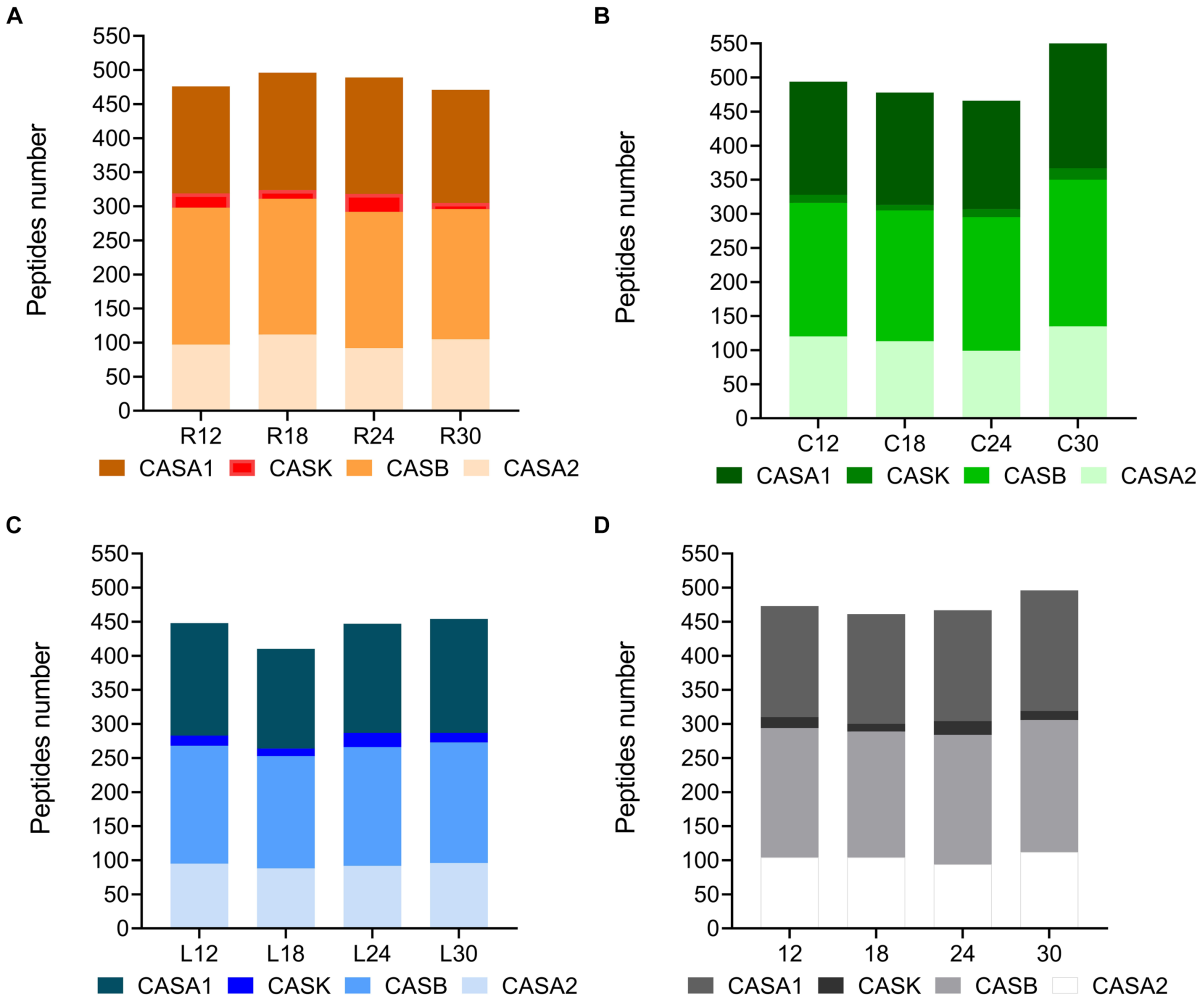
Number of peptides per protein in PR cheeses at different ripening times. Analysis was carried out on low-molecular-weight peptide fractions obtained by ultrafiltration at 3 kDa from the water-soluble peptide fractions extracted from the different PR cheeses. **(A)** Number of peptides per protein identified in PR cheeses from dairy C (orange) at 12, 18, 24, and 30 months of ripening. **(B)** Number of peptides per protein identified in PR cheeses from dairy L (green) at 12, 18, 24, and 30 months of ripening. **(C)** Number of peptides per protein identified in PR cheeses from dairy R (blue) at 12, 18, 24, and 30 months of ripening. **(D)** Number of peptides per protein averaged according to the ripening time. The complete list of identified peptides can be found in Supplementary Table S6.

The ultra-high performance liquid chromatography high-resolution mass spectrometry (UHPLC/HR-MS) performed on the low-molecular-weight water-soluble peptide extracts (<3 kDa) of 12 different cheese samples revealed a total of 603 unique peptides in PR cheeses from dairy R, 609 in PR cheeses from dairy C, and 527 in PR cheeses from dairy L, respectively. The complete list of the identified peptides, together with semi-quantitative and MS data, is reported in Supplementary Table S6. All the identified peptides are derived from the proteolysis of the principal milk caseins, i.e., β-casein, αS1-casein, αS2-casein, and κ-casein. In all samples, the best source of peptides was β-casein, followed by αS1-casein, αS2-casein, and finally κ-casein (Figures 10A,C). C samples showed the highest number of peptides at 12 and 30 months of ripening (Figure 10A), whereas R samples showed the highest number at 18 and 24 months of ripening (Figure 10C). PR samples from dairy L displayed the lowest number of peptides at any ripening time (Figure 10B). The trend of the peptide number over time differed depending on the dairy, while no substantial differences in the number of peptides as a function of the ripening time were found, with the only exception of C30 (Figure 10D). Anyway, the average number of peptides determined during ripening, considering the three dairies, did not vary significantly from each other (Figure 10D).

As shown in Supplementary Figure S2, for each dairy, samples from different ripening times shared most of the identified peptides, suggesting that there is a consistent pool of stable peptides released during the early stages of ripening that are conserved over time. Moreover, different PR samples at the same ripening time shared most of the identified peptides, indicating that the differences in NSLAB cultivable microbiota as well as in cheese manufacturing practices among distinct dairies apparently did not result in any evident peptide variability (Supplementary Figure S3).

**Figure 11:**
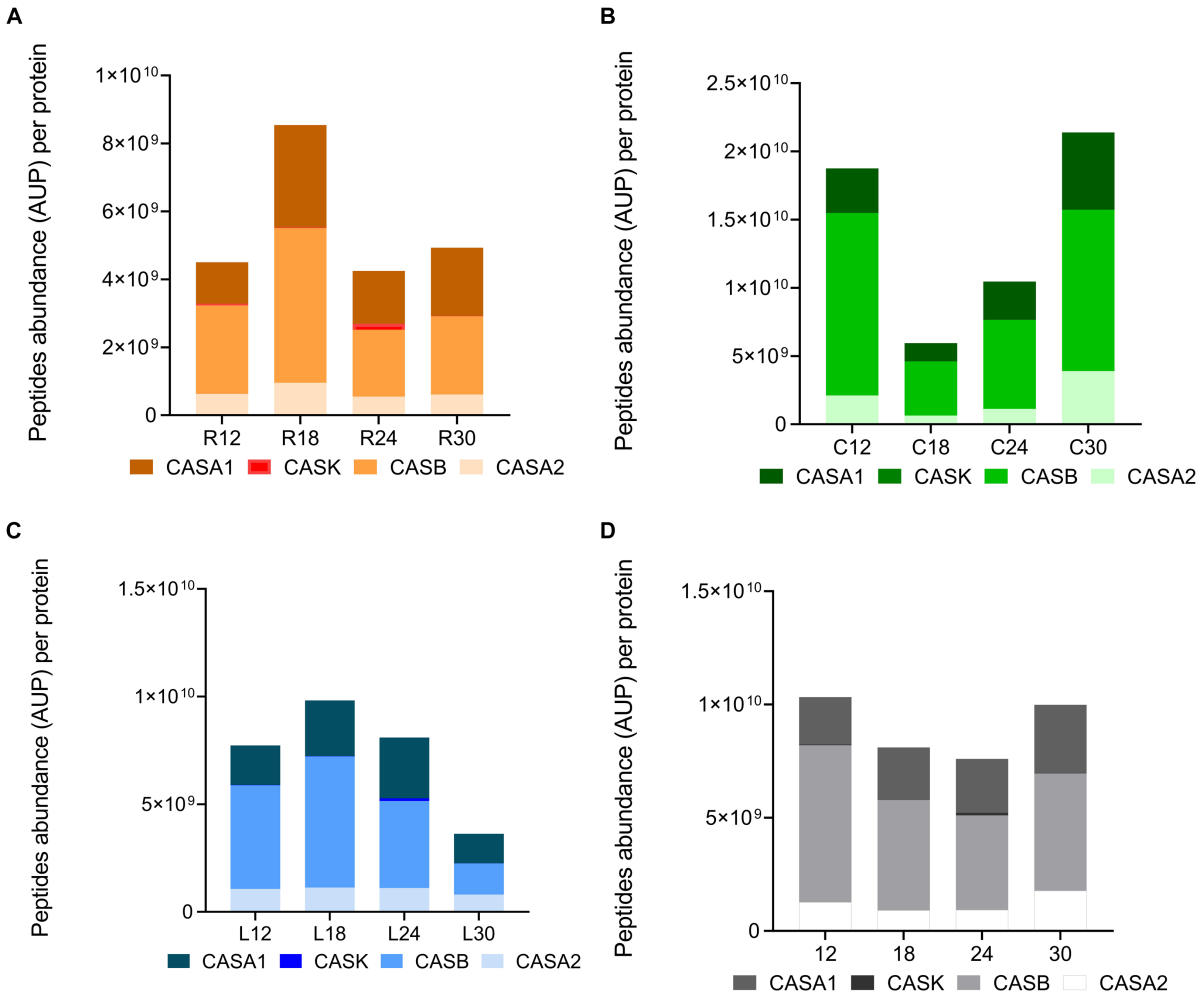
Peptide abundance per protein in PR cheeses at different ripening times. Analysis was carried out on low-molecular-weight peptide obtained by ultrafiltration at 3 kDa from the water-soluble peptide fractions extracted from the different PR cheeses. **(A)** Total peptide abundance per protein in PR cheeses from dairy C at 12, 18, 24, and 30 months of ripening. **(B)** Total peptide abundance per protein in PR cheeses from dairy L at 12, 18, 24, and 30 months of ripening. **(C)** Total peptide abundance per protein in PR cheeses from dairy R at 12, 18, 24, and 30 months of ripening. **(D)** Total peptide abundance per protein averaged according to the ripening time. Data are reported as the sum of the intensity of each identified peptide measured as the area under the peak (AUP) by Skyline analysis. The complete list of identified peptides and the semi-quantitative data can be found in Supplementary Table S6.

To further investigate the effect of ripening time and dairy on peptide profiles, we carried out a semi-quantitative analysis. Data revealed significant differences among the sum of the intensity (peptide abundance measured as the area under the peak for each specific peptide) of the identified peptides in the PR cheese samples. Based on this analysis, significant differences were observed both among dairies and over time (Figures 11A–D). C12 displayed a higher total peptide intensity with respect to R12 and L12 samples (*p* < 0.05). In dairy C, the total peptide abundance strongly decreased by approximately three times from 12 to 18 months of ripening, surging with the highest total peptide intensity at 30 months of ripening (Figure 11A). On the contrary, in dairies L and R, an increase in total peptide intensity from 12 to 18 months of ripening was recorded. Then, in L samples, the total peptide abundance gradually decreased to reach its lowest value in 30-month ripened PR cheese (Figure 11B). Differently, in R samples, the total peptide intensity decreased from 18 to 24 months of ripening, and finally, it reached a plateau (Figure 11C). These differences in total peptide abundance highlighted the role of the different LAB species that colonize the cheeses during ripening in the different dairies on casein hydrolysis.

**Figure 12:**
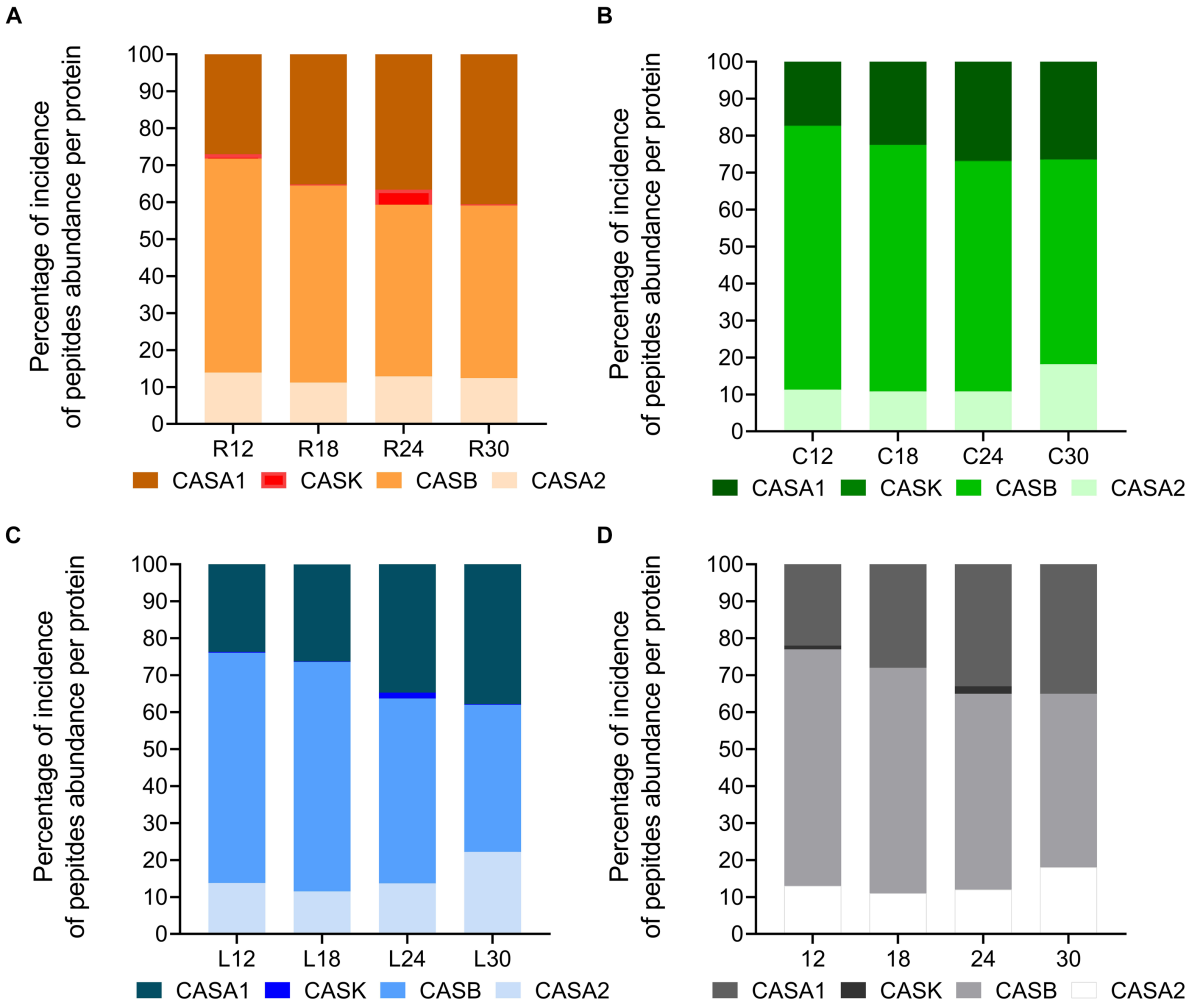
Percentage of incidence of peptide abundance per protein in PR cheeses at different ripening times. **(A)** Percentage of incidence of total peptide abundance per protein in PR cheeses from dairy C at 12, 18, 24, and 30 months of ripening. **(B)** Percentage of incidence of total peptide abundance per protein in PR cheeses from dairy L at 12, 18, 24, and 30 months of ripening. **(C)** Percentage of incidence of total peptide abundance per protein in PR cheeses from dairy R at 12, 18, 24, and 30 months of ripening. **(D)** Percentage of incidence of total peptide abundance per protein averaged according to the ripening time.

Considering the total peptide intensity by protein (Figure 11), β-casein and αS1-casein displayed the highest peptide intensity in any sample, with β-casein overcoming αS1-casein at each ripening time. The trend of the total peptide intensity in β-casein and αS1-casein as a function of the ripening time overlapped that of the total peptide intensity for each dairy. Ripening also affected the percentage of incidence of peptide intensity per protein with respect to the total peptide abundance (Figures 12A–D). In fact, the percentage of incidence of the peptide intensity from β-casein continuously decreased during ripening, whereas the percentage of incidence of peptide intensity for αS1-casein increased according to the ripening time in all the dairies (Figures 12A–D). A similar trend was also observed for the percentage of incidence of peptide intensity for αS2-casein in dairies C and L (Figures 12B,C).

The intensity of individual peptides identified in the different dairies was averaged according to the ripening time (Supplementary Tables S7, S8). Some clusters of specific peptides coming from the hydrolysis of the different caseins displayed characteristic behavior. For example, poly-phosphorylated peptides released from the N-terminal region 1–28 of β-casein peaked and showed the highest intensity in the 12- or 18-month ripened PR cheeses, and then, for most of them, the intensity decreased as the ripening process continued. On the contrary, peptides from the region 38–88 of β-casein showed an increasing trend during ripening, reaching the highest intensity after 30 months of ripening. Finally, peptides from the C-terminal of β-casein (from residue 164 to residue 209) were continuously released during ripening, peaking at 24 months.

Concerning αS1-casein, most peptides from the N-terminal region 1–38 peaked after 18 or 24 months of ripening, whereas peptides in the region between the residues 38 and 80 of αS1-casein, rich in phosphorylation sites, constantly increased in intensity during ripening and reached the highest amounts after 30 months. The same considerations can be made for the C-terminal region of αS1-casein (from residue 80 to residue 199), whose peptides were present in a low amount or absent at the beginning of ripening, peaking after 24 or 30 months.

The presence of bioactive peptides in PR cheese samples was ascertained using the Milk Bioactive Peptides Database and considering only peptides with 100% sequence homology with previously identified bioactive peptides. A total of 49 bioactive peptides were detected, considering all the PR cheese samples (Supplementary Table S9). Most of the identified bioactive peptides came from the hydrolysis of β-casein (30 peptides), followed by αS1-casein (12 peptides) and αS2-casein (six peptides), whereas only one bioactive peptide was identified from κ-casein. Only 25 bioactive peptides out of 49 were detected in all the 12 PR cheese samples.

Most of the identified bioactive peptides presented ACE-inhibitory activity (27 peptides), followed by anti-microbial activity (17 peptides). Other identified peptides have been characterized as antioxidant (seven peptides), immunomodulatory (six peptides), anti-inflammatory (three peptides), and DPP-IV-inhibitory (three peptides) compounds. Finally, two peptides were able to inhibit cholesterol solubility; one was anti-cancer, and one poly-phosphorylated peptide was found to promote calcium uptake. Thirteen identified bioactive peptides possessed more than one biological activity.

As reported above, ACE-inhibitory peptides were the most common bioactive peptides identified in PR cheese. Most of them (15 out of 27 ACE-inhibitory peptides) have been found in all the PR cheese samples. A total of five ACE-inhibitory peptides were not identified in any PR cheese sample from the dairy L. Sample L18 also contained the lowest number of ACE-inhibitory peptides (19 peptides), whereas samples C30 and R12 displayed the highest number of ACE-inhibitory peptides (24 peptides).

**Figure 13:**
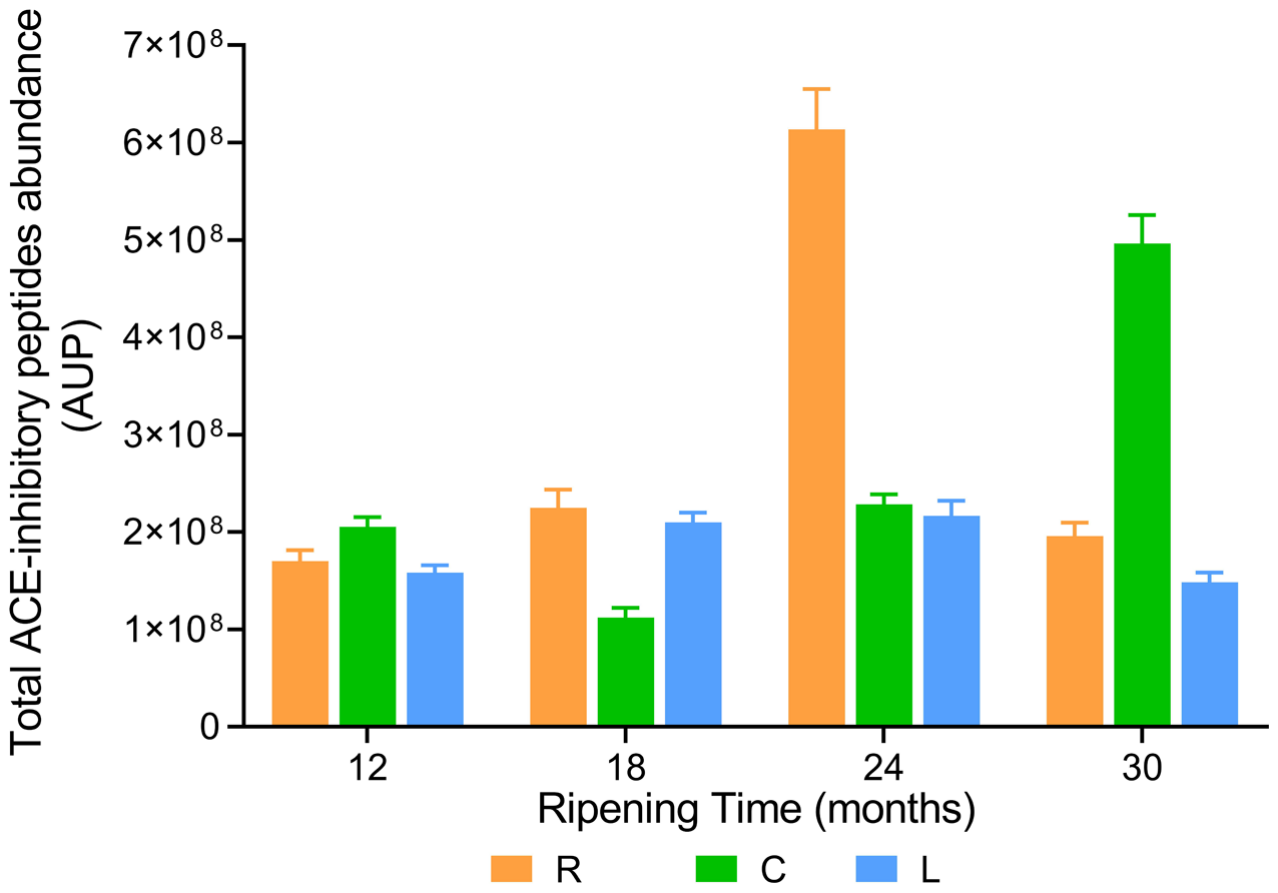
ACE-inhibitory peptide abundance in PR cheeses at different ripening times. Analysis was carried out on low-molecular-weight peptide obtained by ultrafiltration at 3 kDa from the water-soluble peptide fractions extracted from the different PR cheeses (dairy R, orange; dairy C, green; and dairy L, blue). Data are reported as the sum of the intensity of each identified ACE-inhibitory peptide measured as the area under the peak (AUP) by Skyline analysis. The complete list of identified ACE-inhibitory peptides and the semi-quantitative data can be found in Supplementary Table S9.

The evolution of the total ACE-inhibitory peptide abundance during ripening was found to be strongly dairy-dependent, highlighting the role of the different lactic acid bacteria species present in cheese samples (Figure 13). In dairy L, the total ACE-inhibitory peptide intensity increased slightly but significantly from the sample at 12 months of ripening to the sample at 18 months of ripening. Then, it remained almost constant until 24 months of ripening to experience a further decline at 30 months of ripening. Total ACE-inhibitory peptide intensity in sample from dairy R was not significantly different from that of PR cheeses from dairy L at 12 and 18 months of ripening. However, in PR cheese from dairy R at 24 months of ripening, a 2.7 time increase in total ACE-inhibitory peptide abundance was observed. Next, a drastic reduction of approximately three times was recorded in PR sample at 30 months of ripening. PR cheese samples from dairy C behaved differently with respect to the other two dairies. At the beginning of ripening, the total ACE-inhibitory peptide abundance declined, passing from the sample at 12 months of ripening to the sample at 18 months of ripening. As the ripening proceeded, the total ACE-inhibitory peptide abundance started to increase, reaching its highest value at 30 months of ripening.

### 3.7 Effect of ripening time on bioactive peptide patterns

To investigate the effect of ripening time on patterns of bioactive peptides, we first attempted PCA analysis. PCA identified principal components (PC) 1 and 2 as suitable to explain more than 64% of data variability. However, no distinct clusters of samples were found in the score plot (Supplementary Figure S4A). The peptides with the highest loading values were YQEPVLGPVRGP with a positive load for both components 1 and 2, and RPKHPIKHQGLPQEVL with a positive loading for component 1 and a negative loading for component 2 (Supplementary Figure S4B).

**Figure 14:**
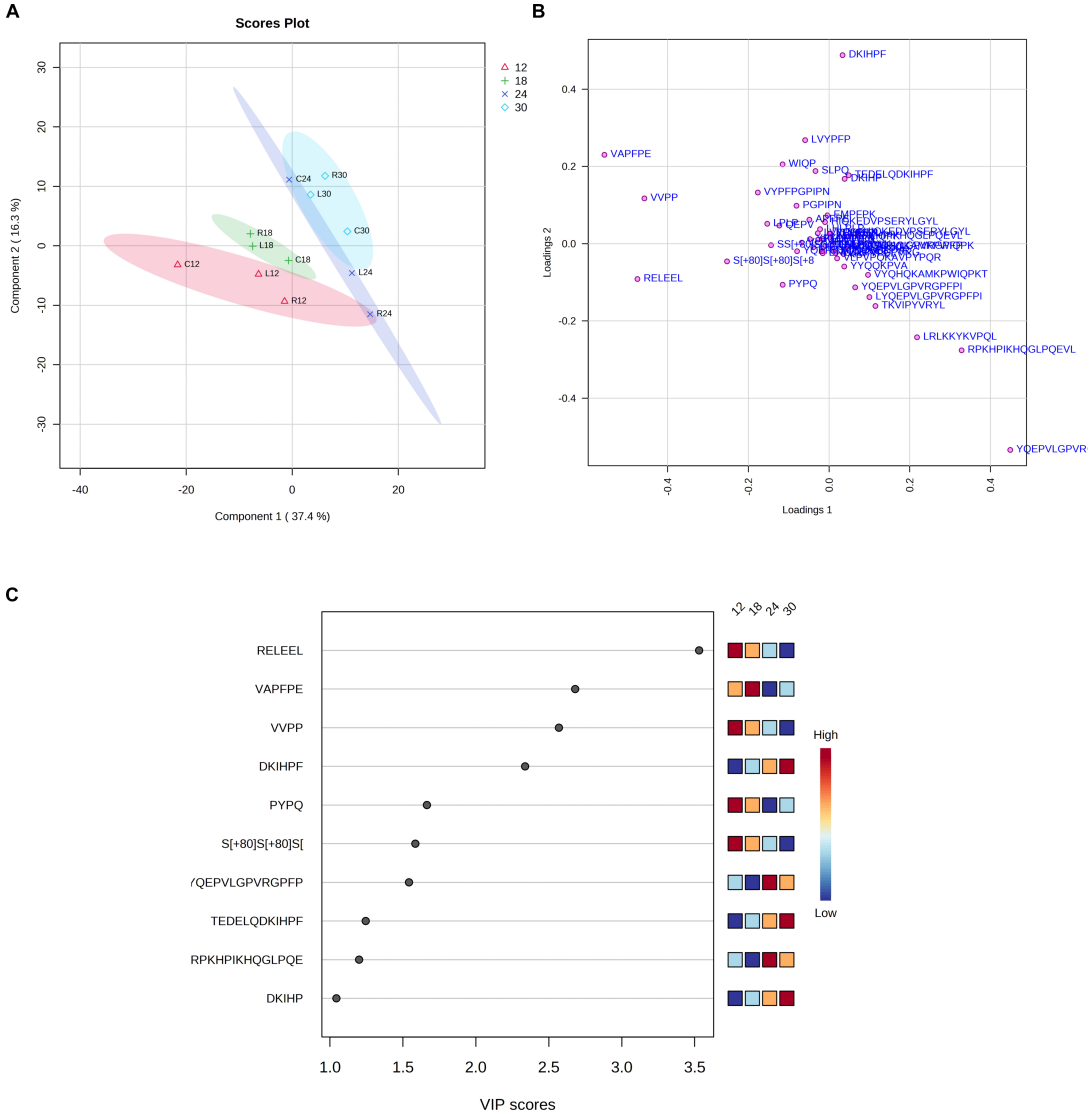
Partial least squares discriminant analysis (PLS-DA) of the bioactive peptide profile from dairies C, L, and R. Scores **(A)** and loadings **(B)** were analyzed at different ripening times (12, 18, 24, and 30 months). **(C)** Bioactive peptides with a VIP score > 1 were found throughout cheese ripening by PLS-DA analysis.

Subsequently, we used the PLS-DA method to classify the samples according to the ripening time. PLS-DA analysis revealed that PR cheese samples can be divided into three clusters based on the first two PCs, explaining 34.7 and 16.4% of the total variance, respectively (Figures 14A,B). PC1 (horizontal plane) was found to have a major weight, resulting in the greatest differentiation of the samples along this plane. Two clusters clearly grouped samples with 18 months and 30 months of ripening, respectively, while the 12-month ripened samples were slightly further apart, especially when looking at PC1. The 24-month group was the most widely dispersed, especially along PC2. This can be explained by the high inter-dairy variability of PR samples, which resulted from a homemade cheese-making process.

The VIP score graph generated by the PLS-DA analysis shows the 10 peptides with a VIP score > 1.0, which mostly contributed to differentiate samples into clusters and were highly present in these samples (Figure 14C). Among these 10 bioactive peptides, we found that the peptides RELEEL (antioxidant peptide), VAPFPE (cholesterol-lowering peptide), VVPP (ACE-inhibitory peptide), DKIHPF (ACE-inhibitory peptide), and PYPQ (antioxidant peptide) displayed the highest VIP values. RELEEL, VVPP, and PYPQ were present in large quantities at the beginning of the ripening process and decreased over time, while DKIHPF, TEDELQSKIHPF (anti-microbial peptide), and DKIHP (ACE-inhibitory peptide) exhibited an opposite trend, with the highest concentration in samples at 30 months of ripening.

### 3.8 Data correlation

The composition, microbiological data, and intensity of the 49 bioactive peptides identified in cheese samples were analyzed by Spearman rank correlations (Supplementary Figure S5). A positive significant correlation (*p* < 0.05) was found between *Lcb. zeae* and eight bioactive peptides, suggesting that the presence of *L. zeae* is pivotal for the release of these bioactive peptides. Most of these peptides were anti-microbial, whereas two had ACE-inhibitory activity. On the contrary, *L. paracasei* was negatively correlated with six out of eight bioactive peptides and was positively correlated with *L. zeae.*

Furthermore, each of the species, *Lcb. Rhamnosus*, *Lcb. Casei*, and *Lcb. Paracasei*, was positively correlated with one specific bioactive peptide (*L. rhamnosus* with the ACE-inhibitory peptide ENLLRF; *Lcb. casei* with the ACE-inhibitory peptide NLHLPLPLL; and *Lcb. paracasei* with the ACE-inhibitory peptide LPLP).

## 4 Discussion

Molecular ecological surveys of the LAB communities have revealed a remarkable degree of bacterial diversity in dairy products. This is especially true for artisanal raw cow milk cheeses, such as PR cheese. Recently, Fontana et al. (2023) found a huge genomic variability in the cheese microbiota, supporting the genetic uniqueness of the strains used in producing different types of PDO cheeses. Local variables on a microgeographical scale, such as temperature and humidity levels, as well as changes in technological parameters and variations in milk quality, ultimately cause fluctuations in the final organoleptic features of the dairy product. Currently, quantitative estimation of these effects is still lacking, and there is a dearth of information on how culturable microbial diversity contributes to biofunctionalities *in vivo*.

This study investigated culturable LAB microbiota in PR cheeses at different ripening times with the aim of establishing a link between cultivable species composition and peptidomic patterns, with a special regard for bioactive peptides. According to PDO guidelines, PR cheese-making entails the practice of back-slopping, where a small portion of the previous batch of sweet whey is fermented to develop the new NWS required for the next fermentation step of raw milk without adding commercial bacterial starters. NWS microbial composition is of great importance in PR cheese-making, as it determines the acidification rate and significantly impacts the overall quality of the final cheese. Three dairies included in this study exhibited remarkable diversity in NWS microbial composition, which significantly affected the subsequent cheese-making steps. Different from the previous studies that reported *S. thermophilus* as a minor species in NWS (Rossetti et al., 2008; Rossi et al., 2012; Santarelli et al., 2013; Morandi et al., 2019), lactococci thermophilic counts were comparable with lactobacilli counts in R and C and even higher than lactobacilli counts in L dairy. Furthermore, lactobacilli counts were generally lower than those found by Gatti et al. (2006) in NWS for Grana Padano cheese under the same culture conditions but comparable with those found by Morandi et al. (2022). Remarkably, *S. thermophilus* was the only species recovered in all three samples. While *L. helveticus* and *S. thermophilus* co-dominate in R and L samples, *L. helveticus* was replaced by *L. delbrueckii* subsp. *lactis* in C. A drastic reduction of *L. delbrueckii* subsp. *lactis* has been recently described in Grana Padano and Trentin Grana NWS (Morandi et al., 2019; Mancini et al., 2021). This species was described as more sensitive than *S. thermophilus* and *L. helveticus* to anti-microbial agents used for equipment cleaning (Morandi et al., 2022). Therefore, dairy-to-dairy differences in cleaning procedures can explain the variable presence of *L. delbrueckii* subsp. *lactis* in NWS. Other reasons for the huge diversity in NWS microbial composition can be related to technological parameters that have changed over space and time, such as temperature-decreasing curves and milk quality. We also consider that NWS microbiota is hard to be cultivated out of the whey environment (Fornasari et al., 2006; Sola et al., 2022). One of the reasons could be the presence of peptides and growth factors in whey, which are lacking in microbiological media. To improve the spectrum of the culturable NWS fraction, we used four different growth conditions. Accordingly, M17 supplemented with SSW mimics a better NWS environment than M17 medium and significantly improves the recovery of streptococci. Another reason for the low cultivability could be the cross-feeding interactions existing in the NWS community, which hamper the microbial growth of axenic cultures (Sola et al., 2022). We cannot exclude that the low cultivability of NWS microbiota could distort the species abundance detected by a culturable approach.

Dissection of the culturable fraction at the strain level confirmed the previous observation that NWS is a complex community composed of several biotypes belonging to a few species (Sola et al., 2022). It is puzzling how this microbial diversity is maintained through several rounds of back-slopping. Recently, phage predation has been recognized as a key evolutionary force driving microbial diversity in natural starter cultures (Mancini et al., 2019; Somerville et al., 2022). The occurrence of several biotypes could make NWS more resilient to phage attacks than dairy starter cultures composed of a few strains or mono-strain dairy starters (as reviewed by Zotta et al., 2022). According to the “kill the winner” model, virulent phages predominantly prey on fast-growing bacteria, thereby suppressing the competitive exclusion of slower-growing bacteria in natural communities (Somerville et al., 2022). This model could explain how NWS diversity remains high over time.

Based on our results, the NWS population was completely replaced by the NSLAB fraction in PR cheese. This confirmed previous studies that reported the replacement of NSLAB over SLAB in the second month of ripening (Solieri et al., 2012; Gatti et al., 2014). Recently, metabarcoding analysis revealed the presence of SLAB in hard-cooked cheeses such as Grana Padano, suggesting that thermophilic NWS microbiota could enter a viable but not cultivable state (VBNC) when pH decreased and lactose was almost completely depleted (Zago et al., 2021). Even if SLAB disappeared by a culture-dependent approach, studies on mesophilic dairy starter suggested that VBNC lactococci are metabolically active in cheese, and their contribution to the cheese flavor increases in a non-growing state, for example, by expressing genes involved in the production of the flavor compounds such as diacetyl and dimethyl disulfide (Decadt and De Vuyst, 2023). While it is well known that lysate SLAB contribute to flavor and support NSLAB growth (Gatti et al., 2014), the presence of viable SLAB in PR cheese as well as the contribution of VBNC SLAB to PR quality have not been investigated yet.

It is well known that SLAB are more sensitive to low a_w_ values than NSLAB. Therefore, low values of moisture and high salt content found in PR samples were expected to inhibit SLAB growth in favor of NSLAB (Beresford et al., 2001). Microbial counts collected from PR cheeses were consistent with previous studies on PR cheese at the same time of ripening (Bottari et al., 2020). As expected, after 30 months of ripening, low a_w_, high salt concentration, and nutrient depletion induce NSLAB cells to die and undergo spontaneous autolysis (Gatti et al., 2014). It has been well established that the main NSLAB present in hard-cooked, long-ripened Grana-type cheeses are *Lacticaseibacillus* spp., followed by *Lactiplantibacillus plantarum* (Gobbetti et al., 2015). In case of raw milk-based cheese, the raw milk microbiota acts as a primary inoculation source, resulting in a higher diversity of NSLAB compared with pasteurized milk cheeses (Vann Hoorde et al., 2010). Other sources of NSLAB are animal rennet and facility equipment, which provide the so-called “in-house microbiota” (Bokulich and Mills, 2013). LCG taxonomy is quite complicated by the high relatedness of species conventionally attributed to this cluster. Recently, the establishment of novel species within the genus *Lacticaseibacillus* further enhanced this complexity (Zheng et al., 2020), and consequently, metabarcoding studies often failed to resolve LCG at the species level (Fontana et al., 2023). Among the 17 species currently attributed to the *Lacticaseibacillus* genus, the most related to LGC is *Lcb. zeae* followed by *Lacticaseibacillus chiayiensis*, a species mainly isolated from meat. Here we developed an improved multiplex PCR assay to fast and accurately discriminate the dairy species *Lcb. casei*, *Lcb. paracasei*, *Lcb. rhamnosus*, and *Lcb. zeae*. Due to this assay, we observed that *Lcb. paracasei* and *Lcb. zeae* were dominant at a long ripening time. To the best of our knowledge, this is the first time that *Lcb. zeae* has been isolated from PR cheese. Even if more samples are required to corroborate this observation, the results suggested that *Lcb. paracasei*, followed by *Lcb. zeae*, could be the most adapted species to live in long-ripened PR wheels. Accordingly, *Lcb. paracasei* has been described as a species well adapted to survive under multiple stresses, i.e., no lactose, low pH, low a_w_ values, and high NaCl concentrations (Neviani et al., 2013). The low presence of *Lcb. rhamnosus* detected in this study partially disagreed with Tagliazucchi et al. (2020) who found *Lcb. rhamnosus* as a dominating species in PR wheels at 12 months of ripening. The high abundance of *Lcb. paracasei* and *Lcb. zeae* species at the highest ripening time could have a great impact on the technological and organoleptic traits of PR cheese, as *Lcb. paracasei* is known to produce several sensorially active compounds (Bancalari et al., 2017; Stefanovic et al., 2018), while *Lcb. zeae* is characterized by high proteolytic aptitude (Vukotić et al., 2016). Proteolysis could contribute to adaptive response to multiple cheese-related stresses, and, accordingly, peptidase activity increases under acidic conditions and carbohydrate depletion (Piuri et al., 2003; Papadimitriou et al., 2016). Similar to *L. helveticus* and *Lactococcus* spp., most dairy *Lacticaseibacillus* strains have CEPs highly efficient in releasing peptides and free amino acids (Solieri et al., 2022). *Lacticaseibacillus* CEPs are less inhibited by the low pH and high salt concentrations compared with lactococcal proteases (Minervini and Calasso, 2022). Among LCG, *L. zeae* strain LMG17315 was reported to possess three copies of CEP-encoding *prt* genes (Vukotić et al., 2016). Based on the previously observed reduction of viable counts over ripening time, we can speculate that CEPs and the intracellular aminopeptidase were released from NSLAB cells into the surrounding cheese matrix, contributing to the peptidomic profile of cheese.

The peptidomic profile of cheese strongly impacts the sensorial features, texture, and biofunctionalities of PR and is strongly related to the composition of SLAB and NSLAB populations. Concerning the general peptide profiles, more than 600 unique peptides were found in cheese samples from dairy C and R and more than 520 unique peptides from dairy L. This study unveiled a higher number of peptides identified in ripened PR cheeses than previously reported. Previous studies identified less than 300 peptides in ripened PR cheeses (Bottari et al., 2020; Martini et al., 2021). Therefore, the current study presents the most detailed evaluation of the PR peptidome to date.

The semi-quantitative analysis of the total peptide intensity (Figures 11, 12) pointed out that β-casein is the preferred hydrolyzed protein by the LAB proteases at the beginning of ripening by promoting the release of peptides at high concentrations, as already suggested by Bottari et al. (2020). Furthermore, as the ripening proceeds, β-casein-derived peptides are further hydrolyzed by LAB proteases into small peptides and/or amino acids or transported inside the cells. The increasing percentages of incidence of the peptide intensity for αS1-casein and αS2-casein during ripening suggest that these proteins are cleaved slowly and with less efficacy by LAB proteases, and their hydrolysis requires a longer ripening time compared with β-casein.

The semi-quantitative analysis of the individual peptide intensity revealed that peptides from the N-terminal region 1–28 of β-casein were easily released at the beginning of the ripening time. Previous studies found that these poly-phosphorylated peptides started to be produced during the first month of ripening of PR cheese and reached their maximum amount after 12 months of ripening (Bottari et al., 2020). These peptides are derived from the hydrolysis operated by LAB CEPs at the initial phases of PR manufacturing and ripening. Most identified poly-phosphorylated peptides had K_28_, N_27_, or R_25_ as C-terminal amino acids. Accordingly, CEPs isolated from several LAB, such as *Lcb. casei*, *Lcb rhamnosus*, *L. helveticus*, and *S. thermophilus*, recognize peptidic bonds between K_28_-K_29_, N_27_-K_28_, and R_25_-I_26_ as common cleavage sites (Solieri et al., 2018; Ji et al., 2021). Next, intracellular LAB aminopeptidases may be responsible for the shortening of these peptides at the N-terminus. Some of the smallest identified poly-phosphorylated peptides presented a biphasic behavior. Their intensity was highest at 12 months of ripening, then decreased until 24 months of ripening, and finally increased again in the 30-month ripened PR cheeses. This is probably due to the massive LAB death observed after 30 months of ripening, which resulted in the release of a high number of active aminopeptidases in the cheese.

Differently, the intensity of peptides from the region 38–88 of β-casein continuously increased during ripening. Most of these peptides were precursors for the anti-hypertensive lactotripeptides VPP and IPP, as well as for β-casomorphins. This region contains numerous cleavage site characteristics of CEPs, and therefore, their release is probably a consequence of the action of bacterial CEPs. Long peptides released during the first phases of ripening are further shortened by the action of the CEPs themselves or by intracellular endopeptidases/aminopeptidases (Ji et al., 2021). Similar behavior was observed for peptides from the C-terminal of β-casein. It has been previously reported that most of the characterized LAB CEPs displayed a marked preference for hydrolyzing the C-terminal region of β-casein and can be responsible for the continuous release of these peptides during ripening (Monnet et al., 1992; Lozo et al., 2011; Ji et al., 2021; Solieri et al., 2022).

Regarding the peptides released from the N-terminal portion of αS1-casein, they can be easily liberated during curding and in the first hours after curding, thanks to the action of chymosin, as well as during ripening by lactic acid bacteria CEPs due to the presence of numerous cleavage sites for CEPs (Sforza et al., 2012; Ji et al., 2021; Solieri et al., 2022; Helal et al., 2023). Most of these peptides reached their highest intensity after 18 or 24 months of ripening. Differently, the region between residues 38 and 80 of αS1-casein, rich in phosphorylation sites, has been suggested to be less susceptible to the hydrolysis by CEPs, and these peptides reached the highest amounts after 30 months of ripening (Sforza et al., 2012; Ji et al., 2021; Solieri et al., 2022; Helal et al., 2023). A similar behavior was observed for peptides released from the C-terminal region of αS1-casein, as previously suggested (Sforza et al., 2012; Ji et al., 2021; Solieri et al., 2022; Helal et al., 2023).

Among the identified peptides, 49 had previously demonstrated biological activities, and most of them were ACE inhibitors. Five ACE-inhibitory peptides identified in PR cheese samples were proven to exert *in vivo* anti-hypertensive effects in spontaneously hypertensive rats (SHR). In detail, the αS1-casein-derived peptide AYFYPEL and the β-casein-derived peptides YPFPGPIPN, LHLPLP, LPLP, and KVLPVPQ were able to decrease blood pressure in SHR to values between 7 and 31.5 mmHg (Tagliazucchi et al., 2019). Moreover, the peptides AYFYPEL and YPFPGPIPN were detected in the bloodstream of human healthy volunteers after consumption of pasteurized milk, suggesting their possible effect also in human subjects (Caira et al., 2022).

Chemometric analysis allowed us to define bioactive peptide biomarkers mostly associated with every ripening time and to establish a list of 10 peptides significantly affected by ripening. Correlation analysis also supported the positive relationship between the recovery of *Lcb. zeae* and the occurrence of eight bioactive peptides with anti-microbial and anti-hypertensive activity. This agrees with the above-mentioned proteolytic aptitude of this species. Interestingly, two β-casein-derived peptides, positively associated with *Lcb. zeae*, such as LLYQEPVLGPVRGPFPIIV and YQEPVLGPVRGPFPIIV, displayed low IC_50_ values against ACE (24 and 101 μmol/L, respectively) and have been demonstrated to decrease blood pressure in SHR (Yamamoto et al., 1994). Significantly, these peptides were found in human plasma after the intake of milk or PR cheese (Caira et al., 2016, 2022).

In conclusion, we depicted the interplay between the microbial cultivable fraction, the peptide profile, and the associated biofunctionalities in PR cheese. We demonstrated that the NSLAB cultivable fraction significantly contributes to the release of bioactive peptides in PR. The positive correlation between *Lcb. zeae* and the presence of bioactive peptides with anti-microbial and anti-hypertensive activities suggests that different species patterns can affect the biofunctionalities of PR cheese. In future, the knowledge of parameters affecting a given species pattern can assist in the improvement of bioactive peptide content in cheese. The wide portfolio of SLAB and NSLAB strains isolated from NWS and PR cheese, respectively, could be useful to select anti-hypertensive adjunct cultures for functional fermented food. Finally, the strain collection established here could be used in future studies with synthetic communities in controlled environments to understand how biotic and abiotic parameters affect the observed patterns of microbial and peptidomics diversity.

## Data availability statement

The datasets presented in this study can be found in online repositories. The names of the repository/repositories and accession number(s) can be found in the article/Supplementary material.

## Author contributions

SM: Writing – review & editing, Methodology, Investigation. LaS: Writing – review & editing, Methodology, Investigation, Data curation. AC: Writing – review & editing, Software, Data curation. MC: Writing – review & editing, Investigation. VP: Writing – review & editing, Investigation, Conceptualization. DT: Writing – review & editing, Writing – original draft, Investigation, Funding acquisition, Conceptualization. LiS: Writing – review & editing, Writing – original draft, Visualization, Validation, Supervision, Software, Project administration, Methodology, Investigation, Funding acquisition, Formal analysis, Data curation, Conceptualization.

## Footnotes

^1^ http://www.parmigianoreggiano.com/consortium/rules_regulation_2/default.aspx

## References

[ref1] Adamberg K. Antonsson M. Vogensen F. K. Nielsen E. W. Kask S. Møller P. L.. (2005). Fermentation of carbohydrates from cheese sources by non-starter lactic acid bacteria isolated from semi-hard Danish cheese. Int. Dairy J. 15, 873–882. doi: 10.1016/j.idairyj.2004.07.017

[ref2] Bancalari E. Sardaro M. L. Levante A. Marseglia A. Caligiani A. Lazzi C.. (2017). An integrated strategy to discover *Lactobacillus casei* group strains for their potential use as aromatic starters. Food Res. Int. 100, 682–690. doi: 10.1016/j.foodres.2017.07.066, PMID: 28873737

[ref3] Beresford T. P. Fitzsimons N. A. Brennan N. L. Cogan T. M. (2001). Recent advances in cheese microbiology. Int. Dairy J. 11, 259–274. doi: 10.1016/S0958-6946(01)00056-5

[ref4] Bertani G. Levante A. Lazzi C. Bottari B. Gatti M. Neviani E. (2020). Dynamics of a natural bacterial community under technological and environmental pressures: the case of natural whey starter for Parmigiano Reggiano cheese. Food Res. Int. 129:108860. doi: 10.1016/j.foodres.2019.108860, PMID: 32036924

[ref5] Bettera L. Levante A. Bancalari E. Bottari B. Gatti M. (2023). Lactic acid bacteria in cow raw milk for cheese production: which and how many. Front. Microbiol. 13:1092224. doi: 10.3389/fmicb.2022.1092224, PMID: 36713157 PMC9878191

[ref6] Bokulich N. A. Mills D. A. (2013). Facility-specific “house” microbiome drives microbial landscapes of artisan cheesemaking plants. Appl. Environ. Microbiol. 79, 5214–5223. doi: 10.1128/AEM.00934-13, PMID: 23793641 PMC3753952

[ref7] Bottari B. Felis G. E. Salvetti E. Castioni A. Campedelli I. Torriani S.. (2017). Effective identification of *Lactobacillus casei* group species: genome-based selection of the gene mutL as the target of a novel multiplex PCR assay. Microbiology 163, 950–960. doi: 10.1099/mic.0.000497, PMID: 28721852

[ref8] Bottari B. Levante A. Bancalari E. Sforza S. Bottesini C. Prandi B.. (2020). The interrelationship between microbiota and peptides during ripening as a driver for Parmigiano Reggiano cheese quality. Front. Microbiol. 11:581658. doi: 10.3389/fmicb.2020.581658, PMID: 33133050 PMC7561718

[ref9] Bottari B. Levante A. Neviani E. Gatti M. (2018). How the fewest become the greatest. *L. casei*’s impact on long ripened cheeses. Front. Microbiol. 9:2866. doi: 10.3389/fmicb.2018.02866, PMID: 30524419 PMC6262004

[ref10] Bottari B. Santarelli M. Neviani E. Gatti M. (2010). Natural whey starter for Parmigiano Reggiano: culture-independent approach. J. Appl. Microbiol. 108, 1676–1684. doi: 10.1111/j.1365-2672.2009.04564.x, PMID: 19849773

[ref11] Bove C. G. Angelis M. D. Gatti M. Calasso M. Neviani E. Gobbetti M. (2012). Metabolic and proteomic adaptation of *Lactobacillus rhamnosus* strains during growth under cheese-like environmental conditions compared to de man, Rogosa, and Sharpe medium. Proteomics 12, 3206–3218. doi: 10.1002/pmic.201200157 22965658

[ref12] Caira S. Pinto G. Picariello G. Vitaglione P. De Pascale S. Scaloni A.. (2022). In vivo absorptomics: identification of bovine milk-derived peptides in human plasma after milk intake. Food Chem. 385:132663. doi: 10.1016/j.foodchem.2022.132663, PMID: 35290952

[ref13] Caira S. Pinto G. Vitaglione P. Dal Piaz F. Ferranti P. Addeo F. (2016). Identification of casein peptides in plasma of subjects after a cheese-enriched diet. Food Res. Int. 84, 108–112. doi: 10.1016/j.foodres.2016.03.023

[ref14] Coppola R. Nanni M. Iorizzo M. Sorrentino A. Sorrentino E. Chiavari C.. (2000). Microbiological characteristics of Parmigiano Reggiano cheese during the cheesemaking and the first months of the ripening. Lait 80, 479–490. doi: 10.1051/lait:2000139

[ref15] Cremonesi P. Vanoni L. Morandi S. Silvetti T. Castiglioni B. Brasca M. (2011). Development of a pentaplex PCR assay for the simultaneous detection of *Streptococcus thermophilus*, *Lactobacillus delbrueckii* subsp. bulgaricus, *L. delbrueckii* subsp. lactis, *L. helveticus*, *L. fermentum* in whey starter for grana Padano cheese. Int. J. Food Microbiol. 146, 207–211. doi: 10.1016/j.ijfoodmicro.2011.02.016, PMID: 21377750

[ref16] Czárán T. Rattray F. P. Møller C. O. D. A. Christensen B. B. (2018). Modelling the influence of metabolite diffusion on non-starter lactic acid bacteria growth in ripening Cheddar cheese. Int. Dairy J. 80, 35–45. doi: 10.1016/j.idairyj.2017.12.010

[ref17] da Silva Duarte V. Lombardi A. Corich V. Giacomini A. (2022). Assessment of the microbiological origin of blowing defects in grana Padano protected designation of origin cheese. J. Dairy Sci. 105, 2858–2867. doi: 10.3168/jds.2021-21097, PMID: 35086714

[ref18] Dallas D. Nielsen S. D. (2018). Milk peptidomics to identify functional peptides and for quality control of dairy products. Methods Mol. Biol. 1719, 223–240. doi: 10.1007/978-1-4939-7537-2_15, PMID: 29476515 PMC6205926

[ref19] Dea Lindner J. Bernini V. De Lorentiis A. Pecorari A. Neviani E. Gatti M. (2008). Parmigiano Reggiano cheese: evolution of cultivable and total lactic microflora and peptidase activities during manufacture and ripening. Dairy Sci. Technol. 88, 511–523. doi: 10.1051/dst:2008019

[ref20] Decadt H. De Vuyst L. (2023). Insights into the microbiota and defects of present-day gouda cheese productions. Curr. Opin. Food Sci. 52:101044. doi: 10.1016/j.cofs.2023.101044

[ref21] Edgar R. C. (2004). MUSCLE: multiple sequence alignment with high accuracy and high throughput. Nucleic Acid. Res. 32, 1792–1797. doi: 10.1093/nar/gkh340, PMID: 15034147 PMC390337

[ref22] Ercolini D. (2020). Secrets of the cheese microbiome. Nat. Food 1, 466–467. doi: 10.1038/s43016-020-0131-9, PMID: 37128078

[ref23] Fontana F. Longhi G. Alessandri G. Lugli G. A. Mancabelli L. Tarracchini C.. (2023). Multifactorial microvariability of the Italian raw Milk cheese microbiota and implication for current regulatory scheme. Msystems 8:e0106822. doi: 10.1128/msystems.01068-22, PMID: 36688869 PMC9948735

[ref24] Fornasari M. E. Rossetti L. Carminati D. Giraffa G. (2006). Cultivability of *Streptococcus thermophilus* in grana Padano cheese whey starters. FEMS Microbiol. Lett. 257, 139–144. doi: 10.1111/j.1574-6968.2006.00155.x 16553844

[ref25] Gatti M. Bernini V. Lazzi C. Neviani E. (2006). Fluorescence microscopy for studying the viability of micro-organisms in natural whey starters. Lett. Appl. Microbiol. 42, 338–343. doi: 10.1111/j.1472-765X.2006.01859.x 16599985

[ref26] Gatti M. Bottari B. Lazzi C. Neviani E. Mucchetti G. (2014). Invited review: microbial evolution in raw-milk, long-ripened cheeses produced using undefined natural whey starters. J. Dairy Sci. 97, 573–591. doi: 10.3168/jds.2013-7187, PMID: 24290824

[ref27] Gatti M. De Dea Lindner J. De Lorentiis A. Bottari B. Santarelli M. Bernini V.. (2008). Dynamics of whole and lysed bacterial cells during Parmigiano-Reggiano cheese production and ripening. Appl. Environ. Microbiol. 74, 6161–6167. doi: 10.1128/AEM.00871-08, PMID: 18689516 PMC2565984

[ref28] Gobbetti M. De Angelis M. Di Cagno R. Mancini L. Fox P. F. (2015). Pros and cons for using non-starter lactic acid bacteria (NSLAB) as secondary/adjunct starters for cheese ripening. Trends Food Sci. Technol. 45, 167–178. doi: 10.1016/j.tifs.2015.07.016

[ref29] Griffiths M. W. Tellez A. M. (2013). *Lactobacillus helveticus*: the proteolytic system. Front. Microbiol. 4:30. doi: 10.3389/fmicb.2013.00030, PMID: 23467265 PMC3587842

[ref30] Helal A. Cattivelli A. Conte A. Tagliazucchi D. (2023). Effect of ripening and in vitro digestion on bioactive peptides profile in Ras cheese and their biological activities. Biology 12:948. doi: 10.3390/biology12070948, PMID: 37508379 PMC10376354

[ref31] Ji D. Ma J. Xu M. Agyei D. (2021). Cell-envelope proteinases from lactic acid bacteria: biochemical features and biotechnological applications. Compr. Rev. Food Sci. Food Saf. 20, 369–400. doi: 10.1111/1541-4337.12676, PMID: 33443792

[ref32] Kim E. Yang S. M. Cho E. J. Kim H. Y. (2020). Novel real-time PCR assay for *Lactobacillus casei* group species using comparative genomics. Food Microbiol. 90:103485. doi: 10.1016/j.fm.2020.103485 32336352

[ref33] Kimura M. (1980). A simple method for estimating evolutionary rates of base substitutions through comparative studies of nucleotide sequences. J. Mol. Evol. 16, 111–120. doi: 10.1007/BF01731581, PMID: 7463489

[ref34] Kumar S. Stecher G. Li M. Knyaz C. Tamura K. (2018). MEGA X: molecular evolutionary genetics analysis across computing platforms. Mol. Biol. Evol. 35, 1547–1549. doi: 10.1093/molbev/msy096, PMID: 29722887 PMC5967553

[ref35] Laref N. Belkheir K. (2022). Application of 16S rRNA virtual RFLP for the discrimination of some closely taxonomic-related lactobacilli species. J. Genet. Eng. Biotechnol. 20:167. doi: 10.1186/s43141-022-00448-8, PMID: 36525129 PMC9756238

[ref36] Lazzi C. Turroni S. Mancini A. Sgarbi E. Neviani E. Brigidi P.. (2014). Transcriptomic clues to understand the growth of *Lactobacillus rhamnosus* in cheese. BMC Microbiol. 14, 28–14. doi: 10.1186/1471-2180-14-28, PMID: 24506811 PMC3928093

[ref37] Letunic I. Bork P. (2019). Interactive tree of life (iTOL) v4: recent updates and new developments. Nucleic Acids Res. 47, W256–W259. doi: 10.1093/nar/gkz239, PMID: 30931475 PMC6602468

[ref38] Liu D. D. Gu C. T. (2020). Proposal to reclassify Lactobacillus zhaodongensis, *Lactobacillus zeae*, *Lactobacillus argentoratensis*, and *Lactobacillus buchneri* subsp. silagei as Lacticaseibacillus zhaodongensis comb. nov., Lacticaseibacillus zeae comb. nov., Lactiplantibacillus argentoratensis comb. nov. and Lentilactobacillus buchneri subsp. silagei comb. nov., respectively and Apilactobacillus kosoi as a later heterotypic synonym of Apilactobacillus micheneri. Int. J. Syst. Evol. Microbiol. 70, 6414–6417. doi: 10.1099/ijsem.0.004548, PMID: 33112225

[ref39] Lolli V. Caligiani A. Gachiuta O. Pizzamiglio V. Bani P. (2021). Study on the effect of ensiling process and ruminal digestion on the synthesis and release of cyclopropane fatty acids in cow feeding. J. Agric. Food Chem. 69, 11026–11032. doi: 10.1021/acs.jafc.1c03204, PMID: 34498864

[ref40] Lopez I. Ruiz-Larrea F. Cocolin L. Orr E. Phister T. Marshall M.. (2003). Design and evaluation of PCR primers for analysis of bacterial populations in wine by denaturing gradient gel electrophoresis. Appl. Microbiol. 69, 6801–6807. doi: 10.1128/AEM.69.11.6801-6807.2003, PMID: 14602643 PMC262258

[ref41] Lozo J. Strahinic I. Dalgalarrondo M. Chobert J. M. Haertlé T. Topisirovic C. (2011). Comparative analysis of β-casein proteolysis by PrtP proteinase from *Lactobacillus paracasei* subsp. paracasei BGHN14, Prt R proteinase from *Lactobacillus rhamnosus* BGT10 and PrtH proteinase from *Lactobacillus helveticus* BGRA43. Int. Dairy J. 21, 863–868. doi: 10.1016/j.idairyj.2011.05.002

[ref42] MacLean B. Tomazela D. M. Shulman N. Chambers M. Finney G. L. Frewen B.. (2010). Skyline: an open-source document editor for creating and analyzing targeted proteomics experiments. Bioinformatics 26, 966–968. doi: 10.1093/bioinformatics/btq054, PMID: 20147306 PMC2844992

[ref43] Mancini A. Rodriguez M. C. Zago M. Cologna N. Goss A. Carafa I.. (2021). Massive survey on bacterial–bacteriophages biodiversity and quality of natural whey starter cultures in Trentingrana cheese production. Front. Microbiol. 12:678012. doi: 10.3389/fmicb.2021.678012, PMID: 34194413 PMC8236940

[ref44] Martini S. Conte A. Tagliazucchi D. (2020). Effect of ripening and in vitro digestion on the evolution and fate of bioactive peptides in Parmigiano-Reggiano cheese. Int. Dairy J. 105:104668. doi: 10.1016/j.idairyj.2020.104668

[ref45] Martini S. Solieri L. Cattivelli A. Pizzamiglio V. Tagliazucchi D. (2021). An integrated peptidomics and in silico approach to identify novel anti-diabetic peptides in Parmigiano-Reggiano cheese. Biology 10:563. doi: 10.3390/biology10060563, PMID: 34205680 PMC8234620

[ref46] Mayo B. Rodríguez J. Vázquez L. Flórez A. B. (2021). Microbial interactions within the cheese ecosystem and their application to improve quality and safety. Food Secur. 10:602. doi: 10.3390/foods10030602, PMID: 33809159 PMC8000492

[ref47] Minervini M. Calasso M. (2022). “*Lactobacillus casei* group” in Encyclopedia of Dairy Sciences. eds. McSweeney P. L. H. McNamara J. P.. 3rd ed (Amsterdam, The Netherlands: Academic Press), 275–286.

[ref48] Møller C. D. A. Ücok E. F. Rattray F. P. (2020). Histamine forming behaviour of bacterial isolates from aged cheese. Food Res. Int. 128:108719. doi: 10.1016/j.foodres.2019.108719, PMID: 31955783

[ref49] Monnet V. Ley J. P. Gonzàlez S. (1992). Substrate specificity of the cell envelope-located proteinase of *Lactococcus lactis* subsp. lactis NCDO 763. J. Int. Biochem. 24, 707–718. doi: 10.1016/0020-711x(92)90004-k, PMID: 1592148

[ref50] Morandi S. Battelli G. Silvetti T. Goss A. Cologna N. Brasca M. (2019). How the biodiversity loss in natural whey culture is affecting ripened cheese quality? The case of Trentingrana cheese. LWT 115:108480. doi: 10.1016/j.lwt.2019.108480

[ref51] Morandi S. Cremonesi P. Arioli S. Stocco G. Silvetti T. Biscarini F.. (2022). Effect of using mycotoxin-detoxifying agents in dairy cattle feed on natural whey starter biodiversity. J. Dairy Sci. 105, 6513–6526. doi: 10.3168/jds.2022-21793, PMID: 35840409

[ref52] Neviani E. Bottari B. Lazzi C. Gatti M. (2013). New developments in the study of the microbiota of raw-milk, long-ripened cheeses by molecular methods: the case of grana Padano and Parmigiano Reggiano. Front. Microbiol. 4:36. doi: 10.3389/fmicb.2013.00036, PMID: 23450500 PMC3584316

[ref53] Neviani E. Lindner J. D. D. Bernini V. Gatti M. (2009). Recovery and differentiation of long ripened cheese microflora through a new cheese-based cultural medium. Food Microbiol. 26, 240–245. doi: 10.1016/j.fm.2009.01.004, PMID: 19269563

[ref54] Nielsen S. D. Beverly R. L. Qu Y. Dallas D. C. (2017). Milk bioactive peptide database: a comprehensive database of milk protein-derived bioactive peptides and novel visualization. Food Chem. 232, 673–682. doi: 10.1016/j.foodchem.2017.04.056, PMID: 28490127 PMC5486413

[ref55] O'Leary N. A. Wright M. W. Brister J. R. Ciufo S. Haddad D. McVeigh R.. (2016). Reference sequence (RefSeq) database at NCBI: current status, taxonomic expansion, and functional annotation. Nucleic Acids Res. 44, D733–D745. doi: 10.1093/nar/gkv1189, PMID: 26553804 PMC4702849

[ref56] Papadimitriou K. Alegría Á. Bron P. A. de Angelis M. Gobbetti M. Kleerebezem M.. (2016). Stress physiology of lactic acid bacteria. Microbiol. Mol. Biol. Rev. 80, 837–890. doi: 10.1128/MMBR.00076-15, PMID: 27466284 PMC4981675

[ref57] Papi M. Palmieri V. Bugli F. De Spirito M. Sanguinetti M. Ciancico C.. (2016). Biomimetic antimicrobial cloak by graphene-oxide agar hydrogel. Sci. Rep. 6:12. doi: 10.1038/s41598-016-0010-7, PMID: 28442744 PMC5431354

[ref58] Piuri M. Sanchez-Rivas C. Ruzal S. M. (2003). Adaptation to high salt in Lactobacillus: role of peptides and proteolytic enzymes. J. Appl. Microbiol. 95, 372–379. doi: 10.1046/j.1365-2672.2003.01971.x, PMID: 12859771

[ref59] Randazzo C. L. Liotta L. Angelis M. D. Celano G. Russo N. Hoorde K. V.. (2021). Adjunct culture of non-starter lactic acid bacteria for the production of Provola Dei Nebrodi PDO cheese: in vitro screening and pilot-scale cheese-making. Microorganisms 9:179. doi: 10.3390/microorganisms9010179, PMID: 33467737 PMC7829852

[ref60] Rossetti L. Fornasari M. E. Gatti M. Lazzi C. Neviani E. Giraffa G. (2008). Grana Padano cheese whey starters: microbial composition and strain distribution. Int. J. Food Microbiol. 127, 168–171. doi: 10.1016/j.ijfoodmicro.2008.06.005, PMID: 18620769

[ref61] Rossi F. Gatto V. Sabattini G. Torriani S. (2012). An assessment of factors characterising the microbiology of grana Trentino cheese, a grana-type cheese. Int. J. Dairy Technol. 65, 401–409. doi: 10.1111/j.1471-0307.2012.00844.x

[ref62] Santarelli M. Bottari B. Lazzi C. Neviani E. Gatti M. (2013). Survey on the community and dynamics of lactic acid bacteria in grana Padano cheese. Syst. Appl. Microbiol. 36, 593–600. doi: 10.1016/j.syapm.2013.04.007, PMID: 23791204

[ref63] Settanni L. Moschetti G. (2010). Non-starter lactic acid bacteria used to improve cheese quality and provide health benefits. Food Microbiol. 27, 691–697. doi: 10.1016/j.fm.2010.05.023 20630311

[ref64] Sforza S. Cavatorta V. Lambertini F. Galaverna G. Dossena A. Marchelli R. (2012). Cheese peptidomics: a detailed study on the evolution of the oligopeptide fraction in Parmigiano-Reggiano cheese from curd to 24 months of aging. J. Dairy Sci. 95, 3514–3526. doi: 10.3168/jds.2011-5046, PMID: 22720910

[ref65] Sgarbi E. Bottari B. Gatti M. Neviani E. (2014). Investigation of the ability of dairy nonstarter lactic acid bacteria to grow using cell lysates of other lactic acid bacteria as the exclusive source of nutrients. Int. J. Dairy Technol. 67, 342–347. doi: 10.1111/1471-0307.12132

[ref66] Sola L. Quadu E. Bortolazzo E. Bertoldi L. Randazzo C. L. Pizzamiglio V.. (2022). Insights on the bacterial composition of Parmigiano Reggiano natural whey starter by a culture-dependent and 16S rRNA metabarcoding portrait. Sci. Rep. 12:17322. doi: 10.1038/s41598-022-22207-y 36243881 PMC9569347

[ref67] Solieri L. Baldaccini A. Martini S. Bianchi A. Pizzamiglio V. Tagliazucchi D. (2020). Peptide profiling and biological activities of 12-month ripened Parmigiano Reggiano cheese. Biology 9:170. doi: 10.3390/biology9070170, PMID: 32708820 PMC7408421

[ref68] Solieri L. Bianchi A. Giudici P. (2012). Inventory of non starter lactic acid bacteria from ripened Parmigiano Reggiano cheese as assessed by a culture dependent multiphasic approach. Syst. Appl. Microbiol. 35, 270–277. doi: 10.1016/j.syapm.2012.04.002, PMID: 22626626

[ref69] Solieri L. De Vero L. Tagliazucchi D. (2018). Peptidomic study of casein proteolysis in bovine milk by *Lactobacillus casei* PRA205 and *Lactobacillus rhamnosus* PRA331. Int. Dairy J. 85, 237–246. doi: 10.1016/j.idairyj.2018.06.010

[ref70] Solieri L. Sola L. Vaccalluzzo A. Randazzo C. L. Martini S. Tagliazucchi D. (2022). Characterization of cell-envelope proteinases from two Lacticaseibacillus casei strains isolated from Parmigiano Reggiano cheese. Biology 11:139. doi: 10.3390/biology11010139, PMID: 35053137 PMC8773131

[ref71] Somerville V. Berthoud H. Schmidt R. S. Bachmann H. P. Meng Y. H. Fuchsmann P.. (2022). Functional strain redundancy and persistent phage infection in Swiss hard cheese starter cultures. ISME J. 16, 388–399. doi: 10.1038/s41396-021-01071-0, PMID: 34363005 PMC8776748

[ref72] Stackebrandt E. (2006). Taxonomic parameters revisited: tarnished gold standards. Microbiol. Today 33, 152–155.

[ref73] Stefanovic E. Kilcawley K. N. Roces C. Rea M. C. O'Sullivan M. Sheehan J. J.. (2018). Evaluation of the potential of *Lactobacillus paracasei* adjuncts for flavor compounds development and diversification in short-aged cheddar cheese. Front. Microbiol. 9:1506. doi: 10.3389/fmicb.2018.01506, PMID: 30026739 PMC6041430

[ref74] Tagliazucchi D. Baldaccini A. Martini S. Bianchi A. Pizzamiglio V. Solieri L. (2020). Cultivable non-starter lactobacilli from ripened Parmigiano Reggiano cheeses with different salt content and their potential to release anti-hypertensive peptides. Int. J. Food Microbiol. 330:108688. doi: 10.1016/j.ijfoodmicro.2020.108688, PMID: 32497940

[ref75] Tagliazucchi D. Martini S. Solieri L. (2019). Bioprospecting for bioactive peptide production by lactic acid bacteria isolated from fermented dairy food. Fermentation 5:96. doi: 10.3390/fermentation5040096

[ref76] Tagliazucchi D. Shamsia S. Helal A. Conte A. (2017). Angiotensin-converting enzyme inhibitory peptides from goats' milk released by in vitro gastro-intestinal digestion. Int. Dairy J. 71, 6–16. doi: 10.1016/j.idairyj.2017.03.001

[ref77] Ventura M. O'flaherty S. Claesson M. J. Turroni F. Klaenhammer T. R. Van Sinderen D.. (2009). Genome-scale analyses of health-promoting bacteria: probiogenomics. Nat. Rev. Microbiol. 7, 61–71. doi: 10.1038/nrmicro2047, PMID: 19029955

[ref78] Vukotić G. Strahinić I. Begović J. Lukić J. Kojić M. Fira D. (2016). Survey on proteolytic activity and diversity of proteinase genes in mesophilic lactobacilli. Microbiology 85, 33–41. doi: 10.1134/S002626171601015X

[ref79] Wilkinson M. G. LaPointe G. (2020). Invited review: starter lactic acid bacteria survival in cheese: new perspectives on cheese microbiology. J. Dairy Sci. 103, 10963–10985. doi: 10.3168/jds.2020-18960, PMID: 33010919

[ref80] Wolfe B. E. Dutton R. J. (2015). Fermented foods as experimentally tractable microbial ecosystems. Cell 161, 49–55. doi: 10.1016/j.cell.2015.02.034, PMID: 25815984

[ref81] Xia J. Sinelnikov I. V. Han B. Wishart D. S. (2015). MetaboAnalyst 3.0—making metabolomics more meaningful. Nucleic Acids Res. 43, W251–W257. doi: 10.1093/nar/gkv380, PMID: 25897128 PMC4489235

[ref82] Yamamoto N. Akino A. Takano T. (1994). Antihypertensive effect of the peptides derived from casein by an extracellular proteinase from *Lactobacillus helveticus* CP790. J. Appl. Sci. 77, 917–922. doi: 10.3168/jds.S0022-0302(94)77026-0, PMID: 8201050

[ref83] Zago M. Bardelli T. Rossetti L. Nazzicari N. Carminati D. Galli A.. (2021). Evaluation of bacterial communities of grana Padano cheese by DNA metabarcoding and DNA fingerprinting analysis. Food Microbiol. 93:103613. doi: 10.1016/j.fm.2020.103613, PMID: 32912585

[ref84] Zheng J. Wittouck S. Salvetti E. Franz C. Harris H. Mattarelli P.. (2020). A taxonomic note on the genus Lactobacillus: description of 23 novel genera, emended description of the genus Lactobacillus Beijerinck 1901, and union of Lactobacillaceae and Leuconostocaceae. Int. J. Syst. Evol. Microbiol. 70, 2782–2858. doi: 10.1099/ijsem.0.004107, PMID: 32293557

[ref85] Zotta T. Ricciardi A. Condelli N. Parente E. (2022). Metataxonomic and metagenomic approaches for the study of undefined strain starters for cheese manufacture. Crit. Rev. Food Sci. Nutr. 62, 3898–3912. doi: 10.1080/10408398.2020.1870927, PMID: 33455430

