# Supplementary material for: Cultivable microbial diversity, peptide profiles, and bio-functional properties in Parmigiano Reggiano cheese
Source: Front Microbiol. 2024 Mar 19;15:1342180. doi: 10.3389/fmicb.2024.1342180 (PMC10985727; doi:10.3389/fmicb.2024.1342180)
Supplement: Supplementary file 1 [file Table_1.DOCX]

Supplementary Material

Cultivable microbial diversity, peptide profiles and bio-functional properties in Parmigiano Reggiano cheese

Serena Martini^1^, Laura Sola^2*^, Alice Cattivelli^1^, Marianna Cristofolini^3^, Valentina Pizzamiglio^4^, Davide Tagliazucchi^1^, Lisa Solieri^3^

^1^Nutritional Biochemistry Lab, Department of Life Sciences, Reggio Emilia, Italy

^2^ Microbial Biotechnologies and Fermentation Technologies Lab, Department of Life Sciences, Modena, Italy

^3^Lactic acid bacteria and Yeast Biotechnology Lab (LYB), Department of Life Sciences, Reggio Emilia, Italy

^4^Consorzio del Formaggio Parmigiano Reggiano, Reggio Emilia, Italy

*** Correspondence: Lisa Solieri**lisa.solieri@unimore.it

# Supplementary Figures


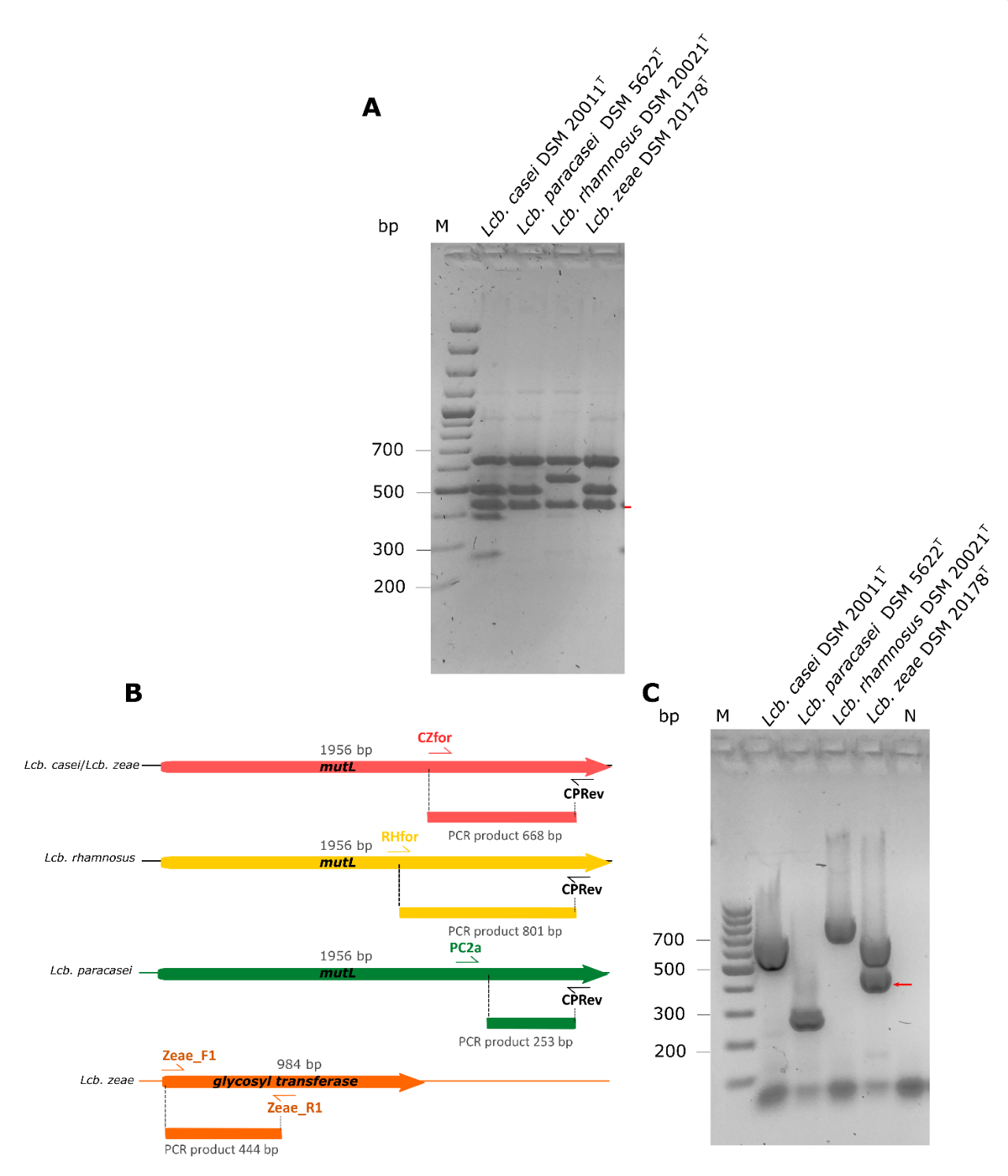


**Figure S1.** Molecular methods for the LCG species attribution. (A) 16S ARDRA pattern obtained with the diagnostic endonuclease *Hha*I for the *Lcb. casei* DSM 20011^T^, *Lcb. paracasei* subsp. *paracasei* DSM 5622^T^, *Lcb. rhamnosus* DSM20021^T^, and *Lcb. zeae* DSM 20178^T^. (B) Strategy overview of improved multiplex PCR assay. (C) Gel electrophoresis analysis of PCR amplicons obtained with the improved multiplex PCR assay. Red arrow indicated double bands of 668 and 444 bp for *Lcb. zeae* DSM 20178^T^. Abbreviation: M, molecular weight marker.


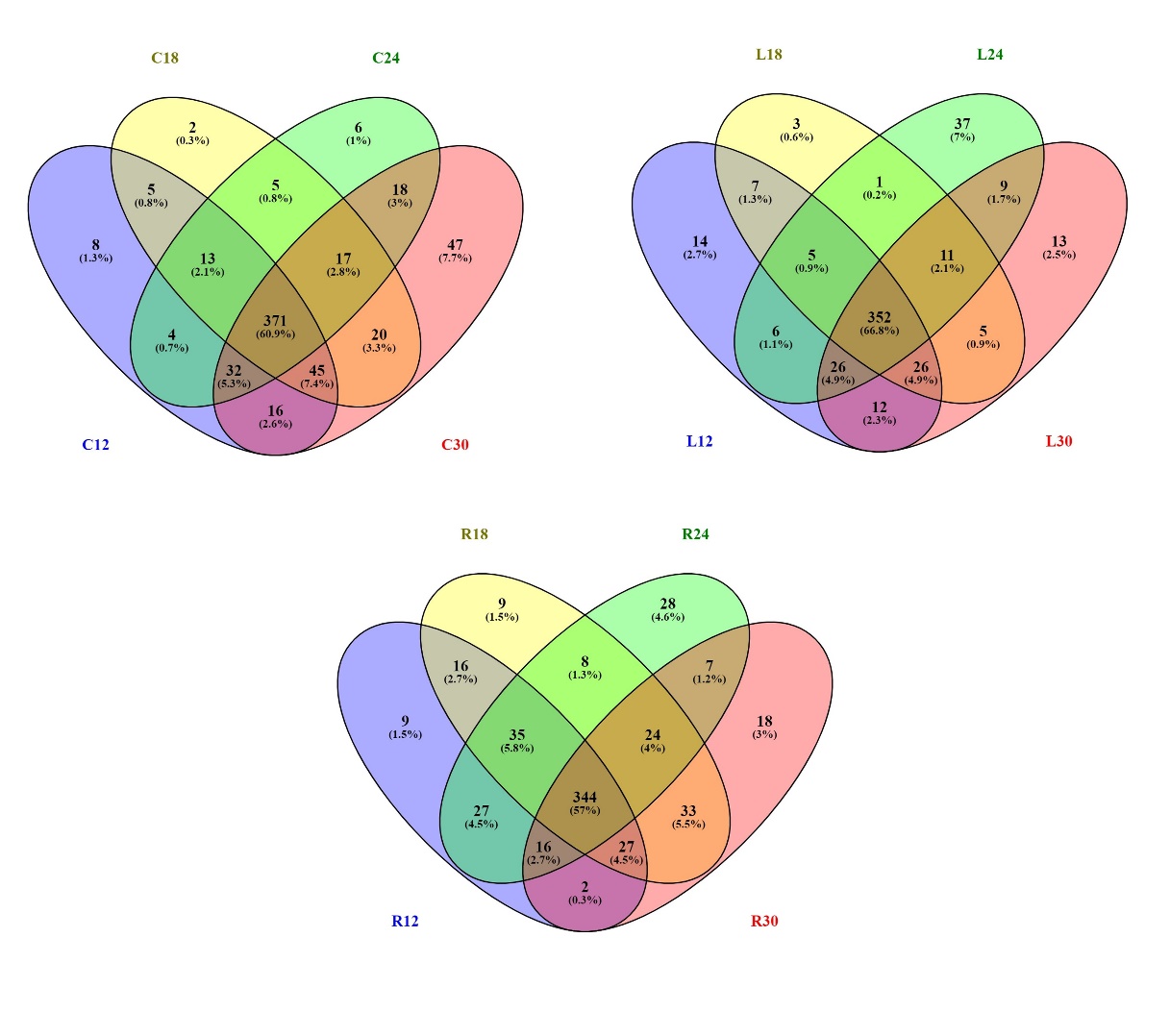


**Figure S2.** Venn diagrams showing differences in peptide profiles of Parmigiano Reggiano cheese samples according to the dairies. The letter C, L and R identify the three different dairies. The number indicates the months of ripening. The complete list of identified peptides can be found in Supplementary Table S6.


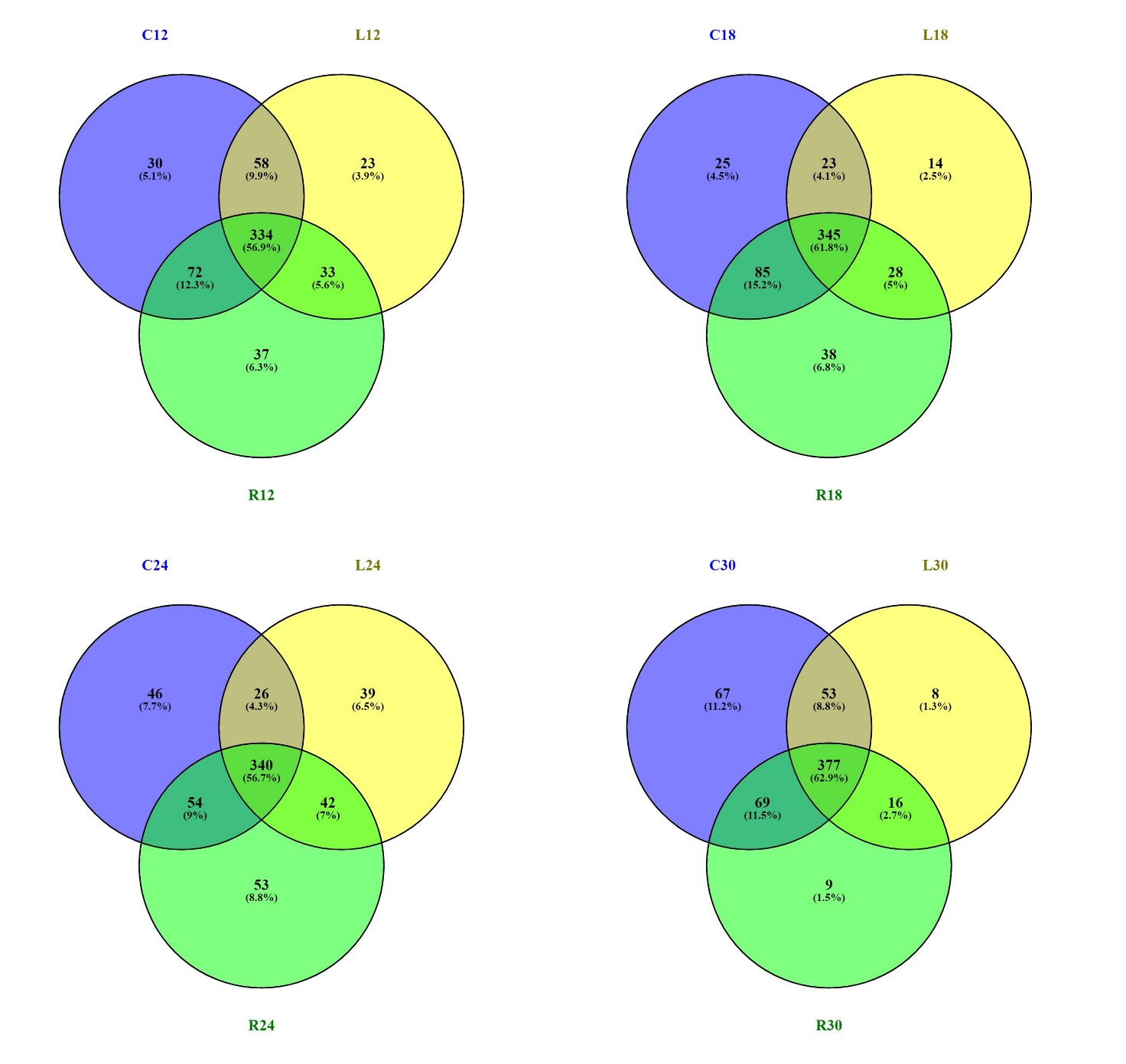


**Figure S3.** Venn diagrams showing differences in peptide profiles of Parmigiano Reggiano cheese samples according to the ripening time. The letter C, L and R identify the three different dairies. The number indicates the months of ripening. The complete list of identified peptides can be found in Supplementary Table S6.


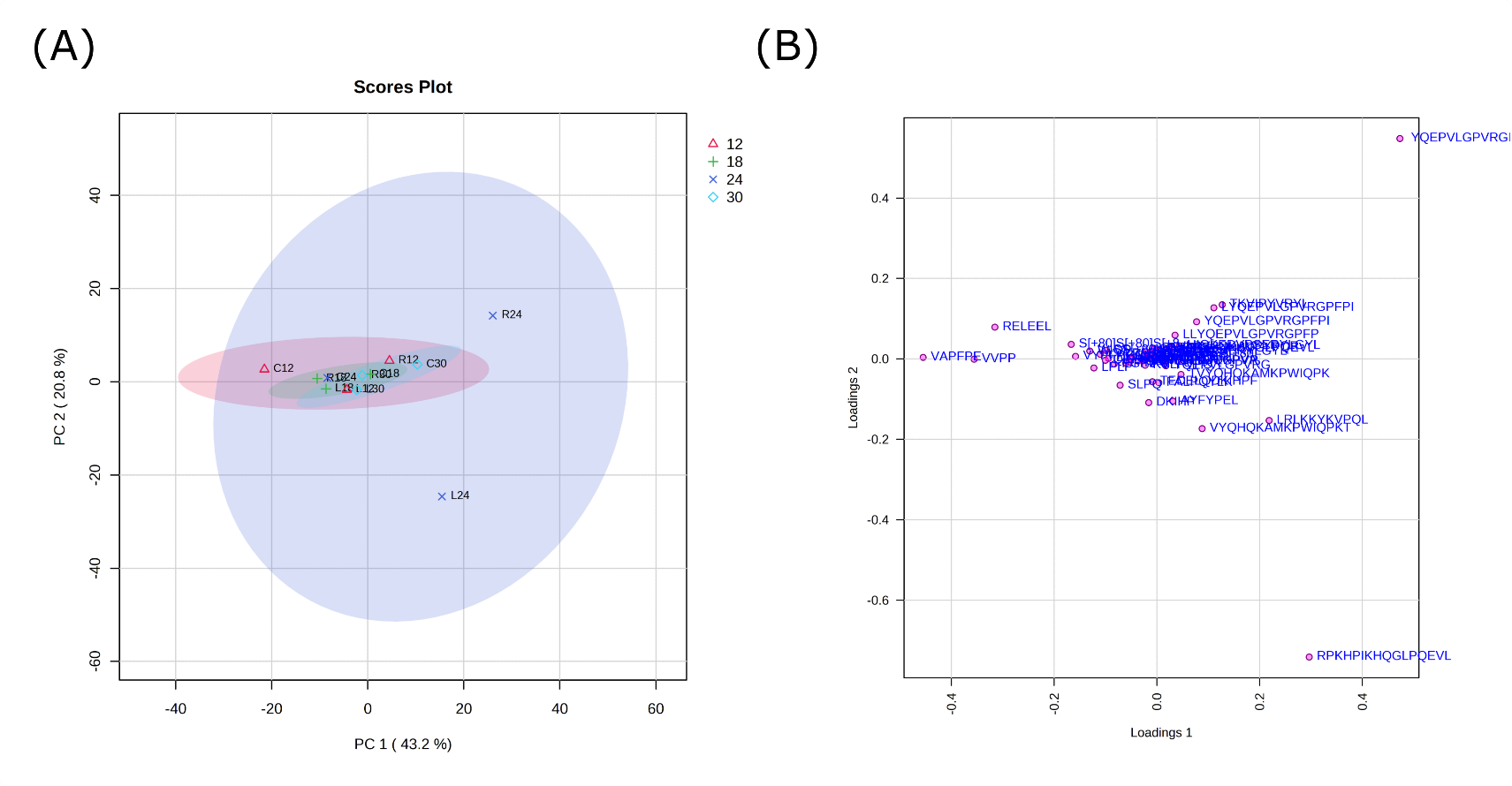


**Figure S4.** Principal component analysis (PCA), including score plot (A) and loading plot (B), of the bioactive peptide profiles from dairies C, L and R at different ripening times (12, 18, 24 and 30 months of ripening).
